# Rizatriptan-Loaded Oral Fast Dissolving Films: Design and Characterizations

**DOI:** 10.3390/pharmaceutics14122687

**Published:** 2022-12-01

**Authors:** Kiramat Ali Shah, Guifeng Li, Lina Song, Binbin Gao, Linyu Huang, Dazhi Luan, Haroon Iqbal, Qingri Cao, Farid Menaa, Beom-Jin Lee, Sulaiman M. Alnasser, Sultan M. Alshahrani, Jinghao Cui

**Affiliations:** 1 College of Pharmaceutical Sciences, Soochow University, Suzhou 215123, China; 2 Department of Pharmacy, Children Hospital of Soochow University, Suzhou 215025, China; 3 School of Pharmacy, Royal College of Surgeons in Ireland (RCSI), D02 YN77 Dublin, Ireland; 4 The Cancer Hospital of the University of Chinese Academy of Sciences (Zhejiang Cancer Hospital), Institute of Basic Medicine and Cancer (IBMC), Chinese Academy of Sciences, Hangzhou 310022, China; 5 Departments of Internal Medicine, Nanomedicine and Advanced Technologies, California Innovations Corporation, San Diego, CA 92037, USA; 6 College of Pharmacy, Ajou University, Suwon 16499, Republic of Korea; 7 Department of Pharmacology and Toxicology, Uniazah College of Pharmacy, Qasim University, Buraydah 52571, Saudi Arabia; 8 Clinical Pharmacy Department, College of Pharmacy, King Khalid University, Abha 61441, Saudi Arabia

**Keywords:** oral disintegrating films, solvent casting method, rizatriptan, pullulan, maltodextrin, propylene glycol

## Abstract

Rizatriptan (RZT) is an efficient anti-migraine drug which belongs to the class of selective 5 HT (1B/1D) serotonin receptor agonists. Nevertheless, RZT elicits several adverse effects and RZT nasal sprays have a limited half-life, requiring repeated doses that could cause patient noncompliance or harm to the nasopharynx and cilia. The current research aimed to develop orally disintegrating films (ODFs) of RZT employing maltodextrin (MTX) and pullulan (PUL) as film-forming polymers, as well as propylene glycol (PG) as a plasticizer. The ODFs were prepared by solvent casting method (SCM). The technique was optimized using Box–Behnken design (BBD), contemplating the ratios of PUL: MTX and different levels of PG (%) as factor variables. The influence of these factors was systematically analyzed on the selected dependent variables, including film thickness, disintegration time (D-time), folding endurance (FE), tensile strength (TS), percent elongation (%E), moisture content (%), and water uptake (%). In addition, the surface morphology, solid state analysis, drug content uniformity (%), drug release (%), and pH of the RZT-ODFs were also studied. The results demonstrated a satisfactory stable RZT-ODFs formulation that exhibited surface homogeneity and amorphous RZT in films with no discernible interactions between the model drug and polymeric materials. The optimized film showed a rapid D-time of 16 s and remarkable mechanical features. The in vitro dissolution kinetics showed that 100% RZT was released from optimized film compared to 61% RZT released from conventional RZT formulation in the initial 5 min. An animal pharmacokinetic (PK) investigation revealed that RZT-ODFs had a shorter time to achieve peak plasma concentration (T_max_), a higher maximum plasma concentration (C_max_), and area under the curve (AUC_0−t_) than traditional oral mini capsules. These findings proposed a progressive approach for developing anti-migraine drugs that could be useful in reducing the complications of dysphagia in geriatric and pediatric sufferers.

## 1. Introduction

Migraine is a debilitating, recurrent neurological illness affecting 1 out of 10 people worldwide [1]. The clinical manifestations of migraine include throbbing headache, nausea, auditory, and olfactory disorders, etc., leading to physical and psychosocial disabilities for individuals. The World Health Organization (WHO) lists migraine as one of the top 20 debilitating medical conditions [2], and it costs more than Alzheimer’s, multiple sclerosis, epilepsy, parkinsonism, and stroke [3]. Nonsteroidal anti-inflammatory drugs (NSAIDs) are most often used to treat mild migraines, while triptans are preferred for severe and chronic migraines [4]. 

Rizatriptan (RZT) is a BCS class III 5-hydroxytryptamine 1B/1D (5-HT1B/1D) receptor agonist with strong hydrophilic nature and limited biofilm penetrability. It constricts brain blood vessels and blocks pain impulses and natural compounds that produce pain, nausea, and other migraine sensations [5]. The suggested adult dose of RZT is 5 mg or 10 mg for immediate management of migraines. Fast-dissolving tablets, traditional tablets, and nasal sprays are commercially marketed. In contrast to other triptans, RZT is frequently used in migraine management due to its faster action and shorter T_max_, which leads to quicker migraine relief [6,7]. Despite its potency, monoamine oxidase (MAO) metabolizes RZT in the liver, resulting in limited bioavailability (40–45%) and a half life of 2–3 h. In addition, traditional RZT tablets have a slow onset of action and migraine symptoms such as upset stomach, vomiting, and nausea, which might impact oral drug absorption [7]. On the other hand, RZT nasal sprays have a limited half-life, requiring repeated doses that could cause patient noncompliance or harm to the nasopharynx and cilia [8]. Thus, a pharmaceutical carrier is needed to overcome the constraint of RZT therapy. 

In recent decades, many studies have demonstrated that oral dispersible films (ODFs) as drug delivery systems (DDS) can provide a rapid onset of therapeutic effects and maximize drug bioavailability. ODFs are conventionally made by solvent casting method (SCM) or hot-melt extrusion (HME) using hydrophilic polymers [9]. Europe has already commercialized RIZAPORT^®^ ODFs prepared by SCM. These ODFs could enhance patient compliance, especially in geriatrics, pediatrics, and individuals with physiological or psychological dysphagia [1]. ODFs acceptability as a drug carrier is further supported by its rapid onset of action, patient adherence, portability, and avoidance of hepatic metabolism [10]. ODFs are flexible, transportable, and effortless to ingest, lowering the danger of choking compared to orally disintegrating tablets (ODTs) [9]. However, ODFs possess significant restrictions owing to compositional variations that might lead to poor mechanical qualities, such as exterior blistering, substrate detaching issues, irregular creases, or fractures. Furthermore, solvent leftovers and extended or altered disintegration and dissolution kinetics restrict ODFs production and clinical implementation of ODFs [11]. Therefore, a thorough study must be undertaken to optimize the formulation parameters of ODFs using experimental design. A variety of hydrophilic synthetic and natural polymers were used as a film forming materials, such as hydroxyl-propyl methyl-cellulose (HPMC) [12], pullulan (PUL) [13], maltodextrin (MLT) [14], Kollicoat^®^ IR [15], cyclodextrins, polyvinyl alcohol (PVA) [1], polyvinyl-pyrrolidone (PVP) [10], and many others.

PUL is a hydrophilic polysaccharide with α-1-4 and α-1-6 glycosidic connections having a molecular weight (MW) of about 200,000 Da and 480 maltotrioses units [16]. The suitability of PUL in ODFs is attributed to its film plasticity, viscosity, hydrophilicity, and degradability. Despite their versatility and unique qualities, PUL-based ODFs have downsides. PUL causes brittleness in films and must be optimized before being used in the design [17]. PUL is pricey owing to its restricted sources. Blending PUL with other low-cost suitable polymers provides an affordable alternative for ODFs formulation with improved physicomechanical properties. Other edible polymeric materials, such as chitosan (CS) [17], HPMC [18], and PVP [19], have recently been co-polymerized with PUL to produce ODFs with better physicomechanical features. This study proposed a compatible and cost-effective MTX as a copolymer with PUL. MTX is hydrophilic dextrin derived from starch. MTX characteristics rely on dextrose equivalent (DE) value. Its MW of 684–6840 Daltons makes it appropriate for ODFs. MTX has the film-forming ability, yet MTX-based ODFs showed in-consistent mechanical properties [20]. Blending PUL with MTX in the ODFs could boost the therapeutic needs of RZT-ODFs for migraine therapy. PUL and MTX have been used as film-forming components. According to our best understanding, the influence of a specific polymeric material or copolymer blend with plasticizers on the physicomechanical characteristics of ODFs designs has been limited. Therefore, developing a new generation of ODFs requires a thorough understanding of the mechanism that affects the polymer blend and plasticizer’s structure correlation. This study aimed to prepare and investigate the effect of formulation parameters (polymers and plasticizer) on the physicomechanical properties and D-time of RZT-ODFs. In-addition, the pharmacokinetic study was performed in rats to compare the pharmacokinetic parameters of optimized formulation (F4) and marketed product after oral administration

## 2. Materials and Methods

### 2.1. Materials 

RZT was bought from Qu Anhui Biotechnology (Suzhou, China). Glycerin (GLY) and PUL were purchased from Aladdin (Shanghai, China). MTX was received from Sigma–Aldrich (St. Louis, MO, USA). PG and polyethylene-glycol (PEG-400) were bought from Sinopharm Biochemical Reagent Co., Ltd. (Beijing, China) and Fisher Scientific Worldwide (Shanghai, China), respectively. RZT^®^ commercial tablets were obtained from Hubei OULY Pharmaceutical Co., Ltd. (Huangshi, China). All additional analytical-grade reagents were purchased locally and utilized as supplied.

### 2.2. Fabrication of RZT-ODFs

RZT-loaded ODFs were produced with minor adjustments to our previously reported process [15]. The SCM was employed to prepare RZT-ODFs. Briefly, accurately weighed (Ohaus Instruments Co., Ltd., Shanghai, China) polymers at varied weight proportions (PUL = 300 to 500 mg; MTX = 0 to 100 mg) were added to 5 mL deionized water (DI) containing different plasticizer concentrations (15 to 30%). The mixture was stirred for two h at a constant speed (2000 rpm) using a magnetic stirrer (model: SH-6/SH-7, Huanghua, China) at room temperature (RT) to dissolve the materials thoroughly. Individually, RZT (50 mg), citric acid (48 mg), aspartame (24.3 mg), and mannitol (24.3) were mixed in 5 mL DI water under constant stirring and temperature until a clear liquid was obtained. The drug solution was poured slowly into the polymer solution and stirred for 1 h to get a homogenized solution. The clear, homogenized mixture was left for 6 h to eliminate air bubbles. The solution was poured onto a 61 cm^2^ substrate and dried for 24 h at RT. The resulting RZT-ODFs were carefully trimmed to a size of 3 × 2 cm^2^, kept in an aluminium pouch, and placed in a desiccator for further evaluation. Figure S1 depicts the whole formulation procedure.

### 2.3. Experimental Design for RZT-ODFs 

The Box–Behnken experimental design (BBD) was employed using three factors and three levels through Design Expert^®^ software (version-10, Stat-Ease, Inc. Minneapolis, MN, USA) [11]. In this design, each numeric element is varied over three levels. The software presents fifteen experiments for each of the factors being considered. The analysis of variance (ANOVA) table revealed that a polynomial linear equation was the most suitable model to represent the data.

(1) $$Y=b_{0}+b_{1\times 1}+b_{2}X_{2}+b_{3}X_{3}+\cdots +b_{12}X_{1}X_{2}+b_{13}X_{1}X_{3}+b_{23}X_{2}X_{3}+b_{123}X_{1}X_{2}X_{3}$$

Equation (1) shows that *Y* is the selected dependent variables (response); b_1_, b_2_, b_3_, … are the regression coefficients for the factors (independent variables); and X_1_, X_2_, X_3_, … are the coded levels of the associated factors [21]. The concentration of X_1_ (PUL, 300–500 mg), X_2_ (MTX 0–100 mg), and X_3_ (PG, 15–30%) were preferred as independent factors. Y_1_ (film thickness; µm), Y_2_ (Folding endurance; FE), Y_3_ (TS; MPa), Y_4_ (E; %), Y_5_ (water content; %), Y_6_ (water absorption; %) and Y_7_ (D-time; s) were considered dependent variables (Table 1). The independent and dependent variables were statistically analyzed employing Design-Expert software, which proved popular for accomplishing the design of experiments (DOE), producing full-order regression models, and relating the studied independent variables with the dependent variables at a 95% level of significance. Moreover, software was employed to perform an ANOVA. Several statistical features including SS (sum of squares), MSS (mean sum of squares), DF (degrees of freedom), MSD (model standard deviation, F value, *p*-value, R^2^ (determination coefficient), Adj-R^2^ (adjusted determination of coefficient), Pred-R^2^ (predicted determination of coefficient) and press were utilized to analyze the experimental data and statistically fit model. Mathematically created models produced three-dimensional (3-D) response surface plots to forecast the correlations between selected factors and variables. The optimum formulation design space was created to achieve thin and fast disintegrated RZT-ODFs with desirable mechanical properties.

### 2.4. Characterization of RZT-ODFs

#### 2.4.1. Appearance and Morphology

Visual inspection of the film was carried out to determine whether the polymer could form a film that seemed to be thin, transparent, uniform, and flexible. ODFs were classified depending on their colour, transparency, and adhesiveness. Before further examination, all ODFs were sealed in aluminium sachets and kept in a desiccator at RT (25 °C ± 3).

The surface characteristics of pure RZT powder and RZT-ODFs were scanned under an optical field emission of the scanning electron microscope (SEM) (model: JEOL JFC-1100E, Tokyo, Japan), equipped with a digital camera. Before analysis, each film was mounted to a metallic stub with double-sided sticky tape using a sputter coater at 10 mA in a vacuum. The photograph was taken at 1.5 K resolution with an accelerating voltage of 10–15 kV.

#### 2.4.2. Thickness, Weight and Drug Content Uniformity

The ODFs thickness was determined by screw gauge (model: J0006 Screw Micrometer, Leqing, China). The sample size of 3 × 2 cm^2^ was held between the screw gauge poles, and thickness was assessed at five spots (centre and four sides). Each test was repeated six times, and the mean± SD was determined.

Six ODFs were taken from each batch to conduct a weight uniformity test. The mean and standard deviation were calculated from the weights of each sample measured with a digital analytical balance (Ohaus Instruments Co., Ltd., Shanghai, China). 

The drug content uniformity of each ODFs was determined using the HPLC method. Briefly, ODFs (3 × 2 cm^2^) were dissolved in 100 mL artificial salivary fluids (pH = 6.8) and homogenized for 15 min using an ultra-sonication bath (Model KQ-300DE, Kunshan, China). The supernatant was collected by centrifugation at 10,000 rpm (10 min), and 20 μL was loaded into the HPLC system. RZT concentrations were determined using a Shimadzu^®^ (model SPD-15c, Shimadzu Corporation, Kyoto, Japan) HPLC system equipped with a Shimadzu^®^ UV detector (u-2600). The mobile phase consisted of 0.2% (*v*/*v*) Trimethylamine (TEA) in water (attuned to pH 5 using 85% ortho-phosphoric acid) and acetonitrile (ACN) (85:15, *v*/*v*). The flow rate was adjusted to 1 mL·min^−1^ for a total run time of 10 min. An HPLC column (CST, 4.6 × 250 mm, 5 µm, 120 A, Suzhou, China) at 40 °C was utilized. Detection was performed at a wavelength of 260 nm. The injection volume was 20 µL, and Shimadzu^®^ LCMS solution software was employed for data acquisition and processing [11]. The following equation calculated the RZT contents in ODFs.

(2) $$\mathrm{RZT}\;\mathrm{contents}\;\left(\%\right)=\frac{\mathrm{Actual}\;\mathrm{amount}\;\mathrm{of}\;\mathrm{drug}}{\mathrm{theoretical}\;\mathrm{amount}\;\mathrm{of}\;\mathrm{RZT}}\times 100$$

#### 2.4.3. Mechanical Analysis

The folding durability was tested by repeatedly folding each ODFs until it broke or its structure was compromised [22]. The average of three assessments was used to determine the outcome.

The mechanical properties of the film were measured using an Instron testing apparatus (model: UH6430, Beijing, China) and a 50 kg weighted cell. Each sample (2 × 1 cm^2^) was held vertically between two clamps. The upper clamp tugged the films at 100 mm per min while the lower clamp was stationary. Once the film was broken, the following formulae were used to estimate the TS and %E of RZT-ODFs [1]. Each film was measured in triplicate.

(3) $$\mathrm{TS}=\frac{\mathrm{Load}\;\mathrm{force}\;\mathrm{at}\;\mathrm{failure}}{\mathrm{Strip}\;\mathrm{thickness}}\times \mathrm{Strip}\;\mathrm{width}$$

(4) $$\%\;E=\frac{\mathrm{Increase}\;\mathrm{in}\;\mathrm{film}\;\mathrm{dimension}}{\mathrm{Initial}\;\mathrm{film}\;\mathrm{dimension}}\times 100$$

#### 2.4.4. Determination of pH, Moisture Content, and Water Absorption

The ODFs were dispersed in 5 mL of purified water. The pH value of each sample was determined by a calibrated pH meter (model: PH-3CU, Changzhou, China). This investigation was repeated three times, and the average± SD was determined.

Three films were taken from each batch and heated in an oven at 105 °C for two hours before being weighed on a computerized analytical balance. After the films dried, each sample was weighed once more, and the moisture content was determined using Equation (5):

(5) $$\mathrm{Moisture}\;\mathrm{content}\;\left(\%\right)=\frac{\mathrm{initial}\;\mathrm{weight}\;-\;\mathrm{final}\;\mathrm{weight}}{\mathrm{final}\;\mathrm{weight}}\times 100$$

An increase in weight measured film capacity to retain moisture after being stored in a desiccator at 79.5 4% relative humidity and 25 ± 2 °C for 72 h, and moisture absorption was assessed using Equation (6):

(6) $$\mathrm{Water}\;\mathrm{absorption}\;\left(\%\right)=\frac{\mathrm{final}\;\mathrm{weight}-\mathrm{initial}\;\mathrm{weight}}{\mathrm{initial}\;\mathrm{weight}}\times 100$$

#### 2.4.5. Disintegration Time (D-Time) 

D-time was measured by immersing a size of 3 × 2 cm^2^ films in a Petri plate that contained approximately 25 mL of artificial saliva (pH 6.8). The sample solution was maintained in a thermostat shaker (model: KYC-100C, Keda Machinery and Instrument Equipment Co., Ltd., Zhengzhou, China) at 37 ± 0.3 °C and shaken constantly (50 rpm). The period taken for a sample to disintegrate was recorded. The study was done six times, and the mean ±SD was calculated.

#### 2.4.6. In Vitro Drug Dissolution Study

The in vitro drug release study was carried out using a USP basket dissolution apparatus (RCZ-8-B, Shanghai, China) containing 300 mL of simulated saliva (SS) in each dissolution apparatus vessel. The release medium (pH = 6.8) was kept at 37 ± 0.5 °C with a rotation speed of 100 rpm. Each ODFs with a dimension of 2 × 3 cm^2^ was placed in a small metal basket (40 mesh) and kept in a dissolution apparatus vessel. The 2 mL sample was withdrawn at specific intervals (2–30 min). An equal media volume was replaced to maintain a constant bath volume [23]. Samples were centrifuged at 10,000 rpm for 10 min, and the supernatant was collected. Approx. 20 µL was injected into the HPLC system as described in Section 2.4.2. The drug release percentage was calculated and plotted versus time intervals.

### 2.5. Compatibility Test of RZT-ODFs

#### 2.5.1. X-ray Diffractometric (XRD) Analysis

The X-ray diffraction pattern of the samples, i.e., pure drug, MTX, PUL, physical mixture, blank film, and RZT loaded ODFs, were scanned by Rigaku Mercury instrument (model: CCD, Tokyo, Japan) with Cu K-α line of copper (radiation source operated at 45 kV and 40 mA at of 5 to 50° (2 θ) range to confirm the crystal form or crystal form transformation of the materials used in the formulation of the film. The scan temperature was 25 degrees Celsius, and the time was set at five microseconds per minute.

#### 2.5.2. Differential Scanning Calorimetry (DSC) Analysis 

Thermal analysis of the samples was performed by DSC apparatus (model: SKZ1052C-1L, SKZ industrial Co., Ltd., Jinan, Shandong, China). Samples were dehydrated in a vacuum oven to remove all water contents before analysis. Approximately 2–3 mg samples of pure materials or prepared films were accurately weighed and placed in a DSC aluminum pan under a nitrogen atmosphere. The samples were heated from 40 to 250 °C at a heating rate of 20 °C per minute, and the XRD diffraction patterns were recorded.

#### 2.5.3. Fourier Transform Infrared (FTIR) Spectroscopy

The substantial molecular interactions between the drug and film-forming materials were explored through FTIR spectrophotometer (model: Excaliber series UMA-500, Bio-Rad, Hercules, CA, USA) using KBr disk method. Briefly, each sample was mixed with potassium bromide (KBr: sample 1:100) and compressed to obtain KBr pellet. The scanning spectra of the samples were achieved in the range of 500–4000 cm^−1^ at a resolution of 2 cm^−1^ [15].

### 2.6. In Vivo Pharmacokinetic (PK) Studies

#### 2.6.1. Experimental Animals

Sprague–Dawley (SD) rats (180–220 g) provided by the animal care and use committees of Soochow University (Suzhou, China) were utilized. The ethical committee of the college of pharmaceutical sciences (Approval No. SUDA20220407A02) approved the animal research proposal, and the research was carried out in strict accordance with the recommendations published by the college of pharmaceutical sciences. Every attempt was made to reduce the level of suffering inflicted on the animals and restrict the number of employed animals.

#### 2.6.2. PK Experimental Design

The rats were kept in stable condition (12 h light/dark cycle at 23 ± 2 °C) with free access to food and water. Overnight fasted rats were divided randomly into two groups, each containing six rats. Before administering optimized ODFs (F4), 50 µL of DI water was placed into the mouth using a micropipette. The film (1 cm^2^) was sliced in half and placed on the tongue of rats (group 1). As a control, RZT marketed tablets equivalent to the dose of the film were crushed and filled in mini capsule shells (size 9). The prepared capsules were fixed in an applicator and intragastrically administered to group 2 animals. All samples were extracted using the liquid-liquid extraction method. Approximately 0.4 mL blood was extracted from retro-orbital plexus in micro-centrifuge heparinized tubes at 10, 30, 60, 90, 180, 360, and 540 min after treatment and instantaneously centrifuged for 20 min at 5000 rpm. The collected plasma (180 µL) was extracted with 1.8 mL dichloromethane to separate plasma proteins and vortex for 2 min [24]. Following centrifugation (10,000 rpm, 10 min), the organic phase was cautiously shifted to a clean micro-tube for dryness by employing a nitrogen evaporator (Hangzhou Aosheng Instrument Co., Ltd., Hangzhou, China). The collected residues were reconstituted using mobile phase (120 µL) and zolmitriptan (ZMT) (10 µL) of 10 µg·mL^−1^ as an internal standard (Figure S2). A 20 µL sample was introduced into the HPLC apparatus. HPLC instrument and chromatographic conditions were comparable, as discussed in Section 2.4.2. A modified flow rate of 1.5 mL·min^−1^ and wavelength of 225 nm were employed for drug analysis in plasma. Various PK parameters such as the area under the curve (AUC_0−t_), maximum concentration (C_max_), time taken to reach the maximum concentration (T_max_), and mean residence time (MRT) were subsequently determined using WinNonlin^®^ 6.1 PK soft-ware platform (Certara, Princeton, NJ, USA).

### 2.7. Statistical Analysis

Each trial was conducted in triplicate, and the findings were expressed as a mean ± SD. The statistical variances among the results were calculated employing Origin Pro and ANOVA. The student *t*-test was used to statistically assess and compare the PK parameter values between the two groups. When the *p*-value was less than 0.05 or more than 0.05, the difference between the group means was considered statistically significant or non-significant, respectively.

## 3. Results and Discussion

### 3.1. Optimizations of Independent Variables

RZT was integrated in polymers-plasticizer blends using SCM due to ease of formulation and affordable cost. A comparison of HME and SCM techniques shows that ODFs produced by the former approach had a longer disintegrating period than the latter [25]. Indeed, materials selection for ODFs fabrication is crucial, as one component influences the properties of another. In this study two compatible polymeric materials (PUL: MTX) were employed to produce RZT-ODFs with desirable qualities. Initially, different plasticizer concentrations (10–30%) were tested. ODFs with less than 15% or more than 30% plasticizer were brittle, stretchable, and sticky. Plasticizer concentrations of 15–30% were studied further. In addition, RZT is an ideal candidate for ODFs formulations due to its low dose (10 mg), smaller MW (<300 Dalton), stability in water and human salivary secretions, partly ionization at oral pH, and ability to permeate the oral mucosal membrane [26].

### 3.2. Box–Behnken Experimental Design (BBD)

A 15-trial BBD with three factors and three levels was chosen to fabricate and optimize RZT-ODFs (Table 1). BBD reduces the number of experiments (15 runs of BBD vs. 27 runs of full factorial design) required to sustain higher-order surface response [27]. This experimental design investigates two or more independent factors and their combined effect on a single response. A 3-factor, 3-level architecture facilitated polynomial regression and quadratic formula employing Design Expert software. The varying quantities of three independent factors such as PUL (X_1_), MTX (X_2_), and PG (X_3_), were identified before the experimental design was implemented. Polymeric mixtures of PUL: MTX (3:0–5:1) were utilized for ODFs to combine the film-producing capabilities of PUL and the high solubility of MTX. All responses, including film thickness Y_1_ (film thickness; µm), Y_2_ (Folding endurance; FE), Y_3_ (TS; MPa), Y_4_ (E; %), Y_5_ (water content; %), Y_6_ (water absorption; %) and Y_7_ (D-time; s) were considered dependent variables and subjected to polynomial linear regression. Software based on central composite design (CCD) and response surface methodology (RSM) depicted an empirical relationship between responses and independent variables. The ANOVA table assumes fixed, non-random components and a quasi-design crossing. A lower probability > F value (*p*-value less than 0.05) indicates that the model terms substantially affect the responses. The adj-R^2^, which is corrected for the number of parameters in the model, is a measurement of the percentage variability around the mean described by the implemented approach. If more model terms do not contribute meaningfully to the model, the adj-R^2^ drops as the number of model terms rise. The anticipated R^2^ is the degree of variation in new data that can be attributed to the model. The Pred-R^2^ and the Adj-R^2^ should be within 0.20, respectively. Otherwise, there could be an issue with the input or the experimental model.

### 3.3. Characterization of RZT-ODFs

#### 3.3.1. Appearance and Morphology

The film-forming capacity of polymers and the physical appearance of all formulations were visually examined, ensuring transparency, bubbles-free, and a smooth surface (Table S1).

**Figure 1:**
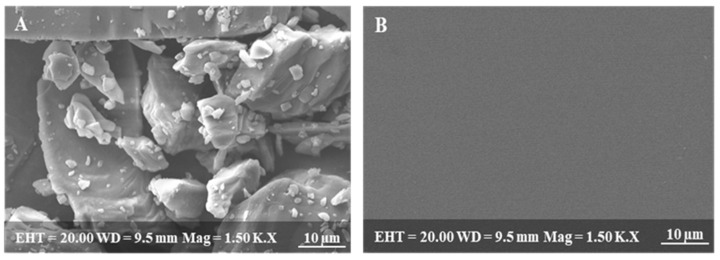
Scanning electron microscopic images of (**A**) pure drug, (**B**) RZT-ODFs.

SEM photograph of pure RZT powder reveals a stable crystalline nature, as shown in Figure 1A [6]. In contrast, the SEM image of RZT-ODFs reveals a homogeneous surface devoid of cracks or transverse ridges (Figure 1B). The findings suggested adequate miscibility and a consistent dispersion of RZT throughout the ODFs [28].

#### 3.3.2. Thickness, Weight, and Drug Content Uniformity

The average thickness of RZT-ODFs was 26.0 ± 4.5 to 87.0 ± 6.6 µm, as shown in Table 1. In all polymer types, thickness values increased sharply (*p* < 0.05) with increasing polymer quantity (Figure S3). This is possibly due to the presence of solid components in the ODFs, which ultimately improved the molecular volume of the RZT-ODFs [29]. In addition, variations in PG contents did not influences film thickness.

The software suggested a multiple regression analysis for response thickness, and the minor standard deviation (SD) values showed less variation around the model (Table 1). Software ensured excellent predictive potential for response film thickness with a high polynomial coefficient (R^2^ = 0.99). The proposed linear model also explained variance in the findings around the median; therefore, the implemented model could account for roughly 96% of the observed variation. Consequently, the model was validated for a comprehensive ANOVA framework, as shown in Table S2. The higher design F value (425.61) and regression coefficients with *p*-values less than 0.05 showed that the Y_1_ model was statistically significant.

Film thickness (Y_1_) was substantially affected by the independent variables (X_1_, PUL, and X_2_, MTX). The Prob > F values of 0.8389 suggested that regression coefficients for PG were not significant relative to the pure error. The F-value of 0.043 signifies that the lack of fit was substantial. A statistical model was created using the ANOVA results that showed a good relationship between factors and responses. Film thickness (Y_1_) was linked with responses (X_1_, X_2_, and X_3_) to generate a final Equation (7).

(7) $$Y_{1}=-39.33+0.21X_{1}+0.20X_{2}-0.022X_{3}\;$$

According to Equation (7), X_1_ and X_2_ exhibited a symbiotic effect on thickness; however, the level of X_3_ revealed a negative impact, indicating that a minor increase in the PUL and MTX quantities substantially increases the thickness of RZT-ODFs. The minus symbol specifies that the amount of PG negatively impacted the thickness of RZT-ODFs. Based on the equation shown above, it is clear that the influence of X_1_ and X_2_ on the response (Y_1_) was considerably more significant (*p* < 0.05) than that of X_3_, which had a relatively more minor impact. 

**Figure 2:**
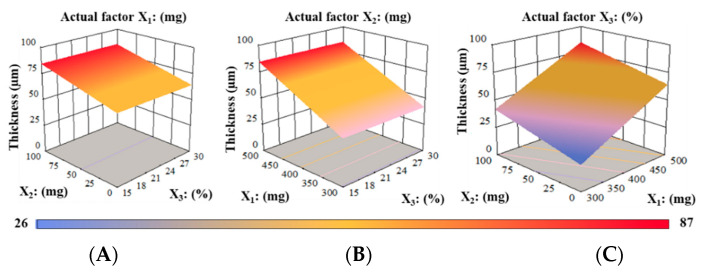
3D surface models describing the influence of (**A**) PUL (X_1_), (**B**) MTX (X_2_), and (**C**) PG (X_3_) on film thickness (Y_1_) of RZT-ODFs.

A contour plot coupled with a three-dimensional (3D) response surface design depicts the influence of PUL (X_1_), MTX (X_2_), or PG (X_3_) on the thickness (Y_1_) of RZT-ODFs, as shown in Figure 2. It is evident from the graphs that RZT-ODFs comprised a substantial level of X_1_ (PUL = 500 mg), with any amount of MTX (X_2_), and PG (X_3_) had a film thickness that ranged from 66.4 to 87.0 µm (Figure 2A). RZT-ODFs formulated with a fixed quantity of MTX (X_2_:100 mg) in a circumstance when the X_1_ level enhanced from a low to a high level dramatically improved the film thickness (Figure 2B). Moreover, a high PG (X_3_) percentage had a negligible influence on the film thickness, as displayed in Figure 2C. Nevertheless, ODFs composed of a low level of PUL (X_1_), a high level of MTX (X_2_), and PG (X_3_) showed a substantial decrease in film thickness to 44.2 µm, as shown in Figure 2.

The influence of polymer and plasticizer amounts on the physicochemical parameters of RZT-loaded ODFs is presented in Table 1. The average weight of RZT-ODFs ranged from 28.1 ± 4.7 to 78.0 ± 4.3 µm, respectively. These values increased significantly (*p* < 0.05) as the polymer amount increased, regardless of polymer type. This is due to solid components, which increase the overall molecular volume of the ODFs [29]. The low ±SD values provide proof of the thickness and weight consistency of the developed formulations. All formulas were non-sticky, transparent, and had a uniform surface (Table S1).

According to the published evidence, the 85–115% limit for therapeutic content uniformity is considered appropriate. The amount of RZT in each ODFs ranged within the defined range of 97.0 to 102.4%, demonstrating that the drug was distributed consistently among all formulae, which complies with USP standards (Table S1).

#### 3.3.3. Mechanical Properties

##### Folding Endurance (FE)

The physical strength of the produced formulations was determined by their FE, which ranged from 64.7 ± 7.8 to 243.3 ± 7.5, respectively. The plasticizer and polymer concentrations influence the physicomechanical properties of the, which ultimately affect the FE of the films. For instance, F1 comprised 15% PG showed considerably lower FE compared to F2 (PG 20%), F4 (PG 25%), or F5 (PG 30%) at fixed proportion of polymeric materials. It is attributed to the electrostatic force amongst the polymers and PG molecules that were not strong enough to overcome the hydrogen bonding interactions due to the low level of PG in RZT-ODFs [30]. The influence of plasticizer on the folding endurance is shown in Figure S4. Similarly, an increase in the polymeric ratio affected the FE of the produced formulations. Higher folding endurance was observed in composite films prepared with a higher proportion of PUL: MTX (500:100 mg) compared to films made with a lower polymer ratio (300:100 mg) or (400:100) at a comparable amount of plasticizers (Figure S5). The impact of factor variables on dependent variables FE was X_3_ > X_2_ > X_1_. Table S3 shows the model summary and ANOVA. The variance between Pred-R^2^ (0.8655) and Ad-R^2^ (0.9073) values was less than 0.2%, indicating that the model was significant. Adequate precision is indicated by an appropriate signal-to-noise ratio. A proportion of more than four is preferred. A current ratio of 22.668 demonstrates a sufficient signal. The design space can be navigated using this approach.The multiple linear regression for the response FE (Y_2_) was represented as follows in Equation (8):

(8) $$Y_{2}=+130.24+29.66X_{1}+2.34X_{2}+52.90X_{3}$$

**Figure 3:**
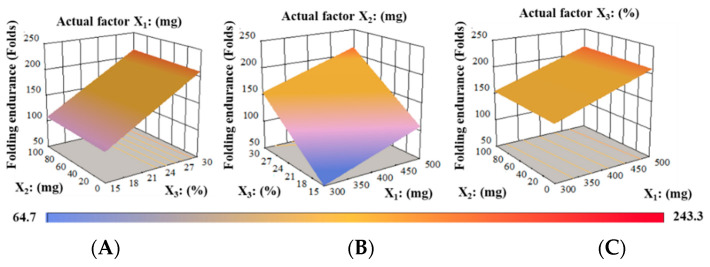
3D surface models describing the influence of (**A**) PUL (X_1_), (**B**) MTX (X_2_), and (**C**) PG (X_3_) on folding endurance (Y_2_) of RZT-ODFs.

Equation (8), expressed in terms of coded factors, can be utilized to anticipate the response for given levels of each factor. By default, the high values of components are encoded as +1 and the low levels as −1. By comparing the factor coefficients, the coded formula can be utilized to determine the relative impact of the components. Equation (8) shows substantial positive influences of all factors on response Y_2_. In addition, higher positive effects were noticed for X_3_ followed by X_1,_ and X_2_, respectively. Accordingly, it was anticipated that increase in the PUL, MTX, and PG concentrations was related to the FE of RZT-ODFs. Figure 3A displays that at fixed X_1_ (PUL), an increase in PG (%) concentration substantially increased FE of the films. Similarly, at a fixed level of X_2_, enhancement in both plasticizer and MTX dramatically increased the FE (Figure 3B). When the X_3_ percentage was maintained constant, the FE substantially increased as the ratio of X_1_ and X_2_ increased. Figure 3C showed that the maximum level of PG (30%) to any polymeric material ratio considerably enhanced the FE of RZT-ODFs.

##### Tensile Strength (TS) Analysis

**Figure 4:**
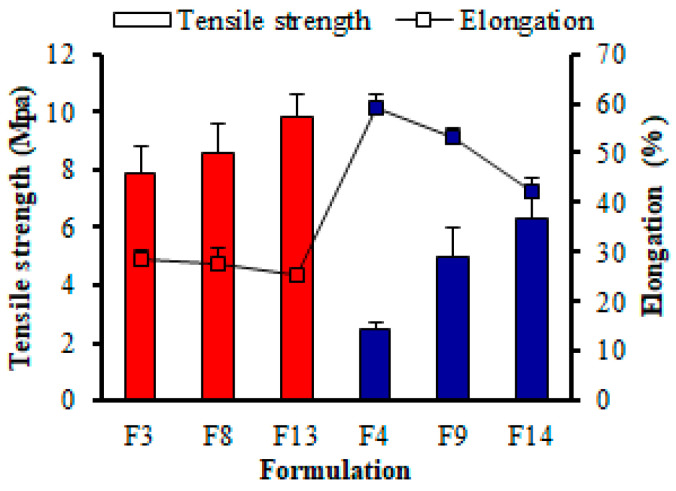
Impact of polymeric material and plasticizers on the mechanical properties of RZT-ODFs formulations. The diagram shows two variables. Tensile strength (white rectangle bar) is represented by the red and blue clustered column; whereas elongation is represented by the black line (white square bar). The *y*-axis for tensile strength is on the left of the figure, while the *y*-axis for elongation is on the right. The common *x*-axis label is formulation codes. Values are shown as mean ± SD (*n* = 3).

The TS analysis demonstrated that as the proportion of MTX and PG to PUL increases, the TS of RZT-ODFs decreases, as shown in Table 1. For instance, RZT-ODFs plasticized with an equivalent amount of PG, only PUL-based films (F3, F8, and F13) showed higher TS and lower %E than the composite formulation of PUL and MTX (F4, F9, and F14), as shown in Figure 4. The results follow the earlier studies [29,31].

The higher DE value MTX in film utilized the mutual influence of PUL: MTX, enhancing the mechanical properties of RZT-ODFs. This is due to the distinct bonding mechanisms between PUL and MTX. The PUL:MTX leading chain segmental elasticity is associated with the chemical arrangement of α-1-6, and α-1-4 bonds. The former is stiffer, whereas the latter has a broader range of elasticity [32]. In addition, the plasticizer content remarkably decreases the TS of the prepared formulation. At the same ratio of polymeric materials, the film comprised of 15% plasticizer (F1) had higher TS than film plasticized with 20% (F2), 25% (F3), and 30% (F5), respectively. This could be attributed to the facile insertion of low MW hydrophilic plasticizer into the polymeric strands, thus preventing the connection between the PUL-MTX strands, increasing intermolecular interaction of the polymer chain, promoting elasticity and minimizing stiffness of RZT-ODFs [33]. Moreover, the software statistically evaluated the factors and their corresponding interaction responses utilizing ANOVA, as shown in Table S4. The Pred-R^2^ of 0.6655 was in reasonable agreement with the Adj-R^2^ of 0.7796; i.e., the difference was less than 0.2. Adequate precision measures the signal-to-noise ratio. A ratio greater than 4 is desirable. The obtained 13.583 indicates a sufficient signal. This model can be used to navigate the design space. The best model F value showed the relevance of the model for Y_3_ (17.50), and regression coefficients with a *p* < value less than 0.0002 showed only a 0.02% chance that a large F-value could occur due to noise. The components PUL (X_1_), MTX (X_2_), and PG (X_3_) had a substantial effect on the response TS (Y_3_). Based on the findings of the ANOVA, a statistical model was developed to outline a workable relationship between the dependent and independent variables.The response Y_3_ (TS, MPa) model produced a final equation regarding coded factors and responses, as shown in Equation (9).

(9) $$Y_{3}=+9.39+2.34X_{1}-1.41X_{2}-6.08X_{3}$$

The TS of RZT-ODFs improved with increasing PUL amounts, as shown by Equation (9), which suggested that PUL (X_1_) amounts positively influenced TS. Data displays that increasing levels of PUL (X_2_) and PG (X_3_) were associated with a decline in response TS and were shown to be most affected by PG (X_3_), followed by MTX (X_2_) and PUL (X_1_), respectively.

**Figure 5:**
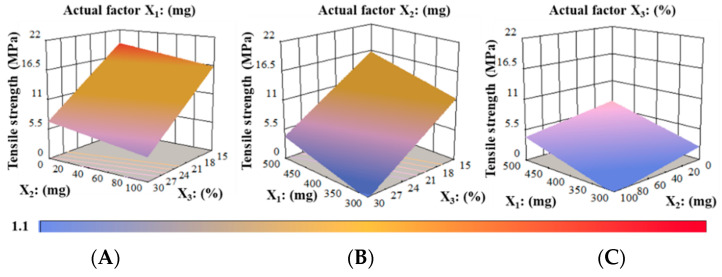
3D surface models describing the influence of (**A**) PUL (X_1_), (**B**) MTX (X_2_), and (**C**) PG (X_3_) on tensile strength (Y_3_) of RZT-ODFs.

The effect of encoded factors on their corresponding response was investigated using 3-D surface plots (Figure 5). The maximum TS of around 21 MPa occurred at a higher level of MTX (X_2_, 100 mg) and a lower level of PG (X_3_, 15%), at a fixed actual factor of PUL (X_1,_ 500 mg) as indicated in Figure 5A. Films comprised varied PG to PUL levels; the TS decreased as the PG to PUL ratio gradually increased (Figure 5B). At a constant PG concentration of 30%, the lowest TS of 1.1 MPa was noticed for the film comprising 300 mg of PUL and 100 mg of MTX, respectively. In addition, PG significantly decreased the TS of RZT-ODFs at a maximum concentration of 30% to any polymeric material proportion, as shown in Figure 5C.

##### Elongation (%) Analysis

The average %E of RZT-ODFs ranged from 13.4 to 62.2%, as revealed in Table 1. The %EE of RZT-ODFs dramatically improve as the concentrations of MTX and PG to PUL rise, and vice versa. The PUL-containing films (F3, F8, and F13) had a lower %E than the composite films of MTX-PUL (F4, F9, and F14), as shown in Figure 4. This could be because PUL-MTX utilizes a diverse range of bonding mechanisms than MTX alone [32]. The PG amount in film (*p* < 0.05) significantly increased the %E. Formulation plasticized with a higher amount of plasticizer (F5, F10, and F15) demonstrated higher flexibility than films comprising low plasticizer levels (F1, F6, and F11). At the equivalent polymer amount, film plasticized with 15% PG (F1, F6, and F11) showed a lower %E than that comprised of 30% PG (F5, F10, and F15). This could be due to introducing low MW and highly hydrophilic PG that increased the polymer chain’s molecular mobility and, in turn, increased the elasticity and decreased the rigidity of the RZT-ODFs [33]. The discrepancy between the pred-R^2^ of 0.8613 and the adj-R^2^ of 0.8894 was less than 0.2, indicating that the two values were reasonably consistent. The signal-to-noise ratio was measured with adequate precision. A value higher than four is preferred. A signal-to-noise ratio of 17.834 indicates a satisfactory signal. This paradigm helped to navigate the design space (Table S5). The Model F-value of 38.53 suggests that the model was significant. A F-value of this magnitude showed just a 0.01% error probability due to noise. Values of Prob > F less than 0.0500 imply that model terms were significant. In this scenario, B and C were ideal model terms. Values larger than 0.1000 means that the model terms were relevant (Table S5). Equation (10) describes the multiple linear regression coefficients for the dependent variable %E (Y_4_).

(10) $$Y_{4}=+27.77-3.60{\;X}_{1}+8.41{\;X}_{2}+23.20{\;X}_{3}\cdots \cdots$$

It is clear from Equation (10) that PUL (X_1_) had an unfavorable effect on Y_4,_ whereas MTX (X_2_) and PG (X_3_) had a favorable impact on the variable. This suggests that even with minor increases in PUL amount in RZT-ODFs, %E was dramatically decreased. Similarly, the positive sign implies that the %E of RZT-ODFs was significantly raised as the amount of MTX and PG were increased. Based on the equation presented above, it is clear that the influence of X_3_ on the response (Y_4_) was more significant (*p* < 0.05) than that of X_2_.

**Figure 6:**
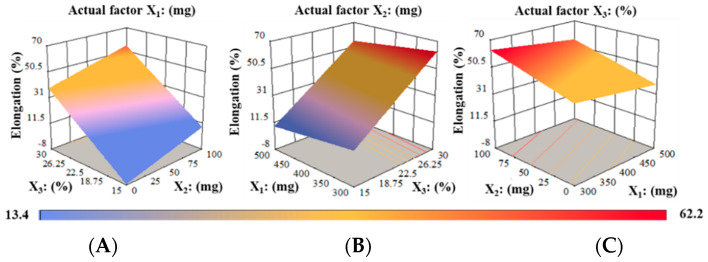
3D surface models describing the influence of (**A**) PUL (X_1_), (**B**) MTX (X_2_), and (**C**) PG (X_3_) on percent elongation (Y_4_) of RZT-ODFs.

The maximum %EE, around 62.2 MPa, was found roughly at the lowest possible PUL level and the maximum PG and MTX levels (Figure 6A). Increases in the ratio of PG to PUL in films resulted in elevated %EE for a given value of the actual factor X_2_ (Figure 6B). Moreover, a contour plot demonstrated that PG, at a maximum of 30% to any polymer ratio (PUL:MTX), significantly improved the %EE of RZT-ODFs, as shown in Figure 6C.

#### 3.3.4. pH, Moisture Content, and Water Absorption

##### pH Determination 

The surface pH of ODFs was evaluated to examine the potential negative consequences of pH changes, as an acidic or alkaline pH may irritate the oral mucosa. The surface pH of all formulations was determined to be in the range of 6.1–7.0, which indicates that they have less potential to damage mucosal surfaces and are consequently more tolerable to individuals (Figure S6).

##### Water Content (%)

**Figure 7:**
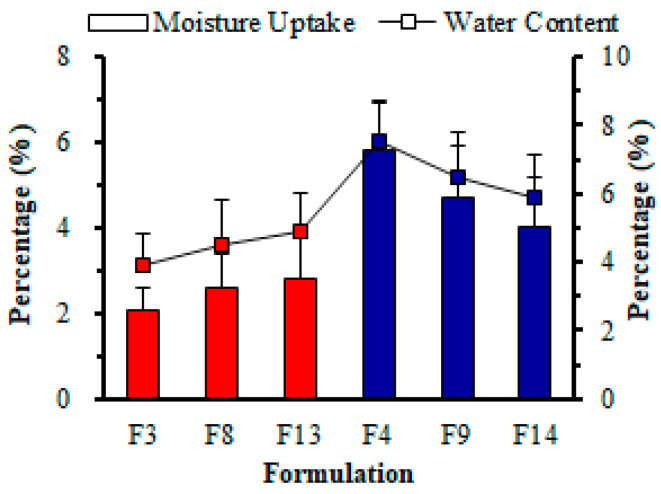
Effect of polymeric materials on moisture uptake (%) and water content (%) of different RZT-ODFs. The diagram shows two variables. Moisture uptake (white rectangle bar) is represented by the red and blue clustered column; wherease water content is represented by the black line (white square bar). The *y*-axis for moisture uptake is on the left of the figure, while the *y*-axis water content is on the right. The common *x*-axis label is formulation codes. Values are shown as mean ± SD (*n* = 3).

Water content in films hindered drying owing to its plasticizing impact. Low water contents induced brittleness, while high water contents supported the adhesion of ODFs. In our investigation, water content (%) ranged from 3.9% to 8.2%, respectively, as shown in Table 1. The composite films of PUL and MTX (F4, F9, and F14) showed significantly more water content than the only PUL-based formulations (F3, F8, and F13) due to the exceptionally hygroscopic nature of MTX (Figure 7). When the ODFs were subjected to 105 °C temperature, formulation F3 composed of a low polymer amount (300 mg), had less water content than F13, which was made of 500 mg PUL. This phenomenon is attributed to the hydrophilic nature of polymeric materials. Additionally, the amount of water increased proportionally as the PG level in the composite ODFs increased (Figure S7). RZT-ODFs plasticized with 15% plasticizer (F1) displayed noticeably lower moisture contents than those with 20% (F2), 25% (F4), and 30% (F5), respectively. The hydrophilic properties of plasticizer could drive this interaction, resulting in a substantial hydrodynamic complex of PUL-MTX-PG, thus enhancing film moisture contents. These findings coincide with those reported earlier [34].

ANOVA statistical analysis was used to comprehend the coded factors on the computed responses, and the results are shown in Table S6. The difference between the Pred-R^2^ of 0.6040 and the Adj-R^2^ of 0.8155 was more significant than 0.2, contrary to what one may often anticipate. This can be a sign of a considerable block effect or a possible issue with a model. Correspondingly, adequate precision measured the signal-to-noise ratio.

A ratio of at least four is preferred. According to recent findings, a ratio of 14.466 suggests a significantly strong signal. The design space can be explored using this model (Table S6). A Model F-value of 21.63 signifies that the model was statistically significant. A noise level of this magnitude would only have a 0.01% probability of producing an F-value of this magnitude. Values of Prob > F that were lower than 0.0500 demonstrated that model terms were significant. In this particular instance, X_1_ (PUL), X_2_ (MTX), and X_3_ (PG) were important model terms. It is possible to utilize the equation expressed in coded factors to predict the response for different levels of each factor. The high levels of the components were coded as +1, while the low levels of the elements were coded as −1. The coded equation can be utilized by evaluating the factor coefficients to determine the influence of the relative component, as shown in the following Equation (11).

(11) $$Y_{5}=+5.29-0.44{\;X}_{1}+1.12{\;X}_{2}+0.78X_{3}$$

Equation (11) shows a linear negative influence for X_1_ and a positive linear impact for X_2_ and X_3_, respectively. In addition, positive effects on the interface amongst X_1_ and X_2_ were noticed for response Y_5_. Thus, it was anticipated that the increase of the MTX and PG concentrations was related to enhance water contents (%) in RZT-ODFs. Moreover, the actual coded amount of PUL (300 to 500 mg) cooperated in decreasing the water contents of the studied formulations.

**Figure 8:**
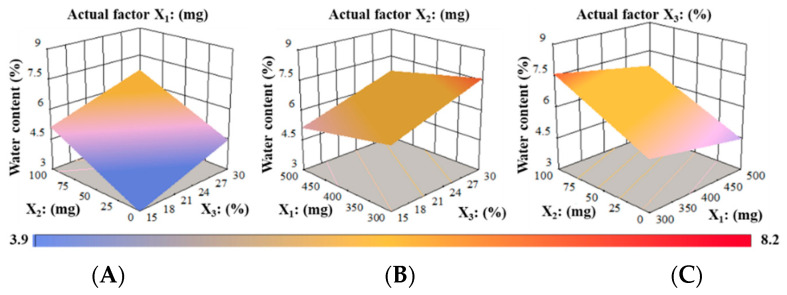
3D surface models describing the influence of (**A**) PUL (X_1_), (**B**) MTX (X_2_), and (**C**) PG (X_3_) on water content (Y_5_) of RZT-ODFs.

The association between the dependent variables and water content is depicted in Figure 8. The water content (Y_5_, %) reduced as the amount of PUL (X_1_) grew; however, water contents increased when the ratio of X_2_ and X_3_ in RZT-ODFs was augmented, as shown in Figure 8A. It was determined that at a given fixed factor of PUL (X_1_ = 500 mg), with any amount of X_2_ (MTX) and X_3_ (PG), the water content ranged from 4.9 to 6.4%, respectively. Furthermore, a high level of water content was seen when the MTX (X_2_ = 100 mg) and PG (X_3_ = 30%) amounts were kept high, and the PUL (X_1_ = 300 mg) quantity was kept low, as indicated in Figure 8B. The considerable oppositional effect of PUL (X_1_) and synergistic effect of MTX (X_2_) were explained by a fixed factor of X_3_ on the water content (%) of RZT-ODFs (Figure 8C). The findings agreed with the software’s linear relationship of water content (%).

##### Water Absorption (%)

Water absorption (%) is a primary indicator of film stability. ODFs are envisaged to have higher water susceptibility as the proportion of hydrophilic polymers and plasticizers increases [35]. The obtained moisture uptake (%) values of RZT-ODFs were 2.1 to 6.1%, respectively, as indicated in Table 1. Furthermore, the concentrations of MTX and PG significantly increased moisture uptake. Figure 7 shows that RZT-ODFs made with only PUL (F3, F8, and F13) had a lower level of water uptake (%) than blended formulations of PUL and MTX (F4, F9, and F13) at a comparable amount of PG (25%). Furthermore, variations in plasticizer percentage impacted moisture absorption (%). RZT-ODFs plasticized with 15% (F6) absorbed a reduced amount of moisture than formulation made of 20% (F7), 25% (F9), and 30% (F10) when an equivalent polymer ratio (PUL: MTX = 400:100) was utilized. The overall tendency to increase moisture uptake (%) with rising MTX (Figure S8) and PG levels was observed in the film. This is attributed to the high MTX mobility, which allows the PG to penetrate the molecular chains of polymeric materials, exposing more of their strands to water absorption [36]. Overall, the results demonstrated that only PUL-based RZT-ODFs absorbed the least amount of moisture due to the plain polymer backbone of PUL, which is devoid of side chains. Hence, the molecular chains in the ODFs were strongly connected, preventing water-content molecules from moving across PUL [37]. Therefore, integrating MTX into PUL is an efficient and effective approach for reducing the fragility of RZT-ODFs. The software recommended a linear regression model for independent variable moisture uptake (%) with lower ±SD values (Table S7). The gap between the pred-R^2^ (0.6139) and the Adj-R^2^ (0.8213) was more prominent than 0.2, suggesting that the two measured values were not closely related as one might expect. This is due to a massive block effect or an issue with the model. The signal-to-noise ratio was determined by adequate precision. A ratio higher than four is preferred. The current signal-to-noise ratio of the model was 14.953 suggests a sufficient signal. This paradigm helps to explore the design space. The Model F-value of 22.45 indicates that the model was statistically significant. There was a 0.01% probability that an F-value of this was statistically significant. Values of Prob > F less than 0.0500 suggests practical model terms (Table S7). PUL and MTX were critical model terms in this instance.

Equation (12) was produced when moisture absorption (Y_6_) was correlated with independent variables (X_1_, X_2_, and X_3_):

(12) $$Y_{6}=+5.29-0.44{\;X}_{1}+1.12{\;X}_{2}+0.78{\;X}_{3}$$

Equation (12) showed that PUL amount (X_1_) suggested a negative influence, while MTX amount (X_2_) and PG amount (X_3_) had a synergistic impact on the response Y_6._ It was noted that slight increases in PUL amount significantly decreased moisture uptake (%). The positive sign specifies that as the level of MTX and PG increased, the moisture absorption (%) of RZT-ODFs substantially increased. Equation (12) shows that the influence of X_2_ on the response (Y_6_) was more substantial (*p* < 0.05) than X_3_.

**Figure 9:**
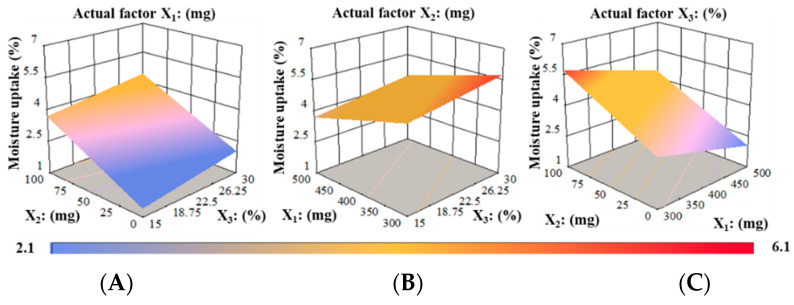
3D surface models describing the influence of (**A**) PUL (X_1_), (**B**) MTX (X_2_), and (**C**) PG (X_3_) on moisture absorption (Y_6_) of RZT-ODFs.

The relationship between the dependent variables and water absorption (%) was further explored using 3-D surface plots. Figure 9 displays the correlation between the factors and response moisture uptake (%). At a fixed actual factor of X_1_ amounts, with increasing in X_2_ and X_3_ amounts, the moisture uptake (%) gradually increased in films (Figure 9A). Correspondingly, as the amount of X_1_ gradually increased, the water absorption (%) of ODFs decreased when the actual factor X_2_ was kept constant. Moreover, the elevation in X_3_ percentage potentially increased moisture uptake of RZT-ODFs, as shown in Figure 9B. At a fixed actual rate of X_3,_ the water absorption rises as the amount of X_2_ in films increases. Similarly, the enhancement in the X_2_ level potentially enhanced the moisture uptake of RZT-ODFs, as shown in Figure 9C.

### 3.4. Disintegration Time (D-Time)

The average D-time of RZT-ODFs ranged from 15.3 to 44.7 s, as shown in Table 1. The D-time analysis revealed that as the amount of polymeric materials increases, the D-time of RZT-ODFs increased.

**Figure 10:**
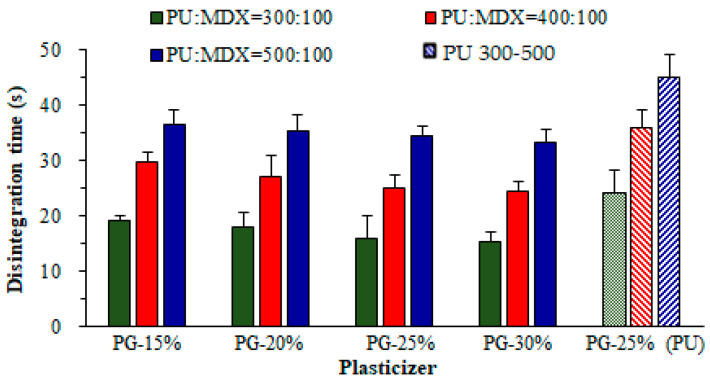
Influence of maltodextrin and propylene glycol amounts on the D-time of RZT-ODFs. Solid filled clustered columns represent; PUL:MTX = 300:100 (green), PUL:MTX = 400:100 (red), and PUL:MTX = 500:100 (blue), whereas pattern filled clustered columns represent only PU based formulations; PU = 300 (50% green), PU = 400 (downward diagonal red), and PU = 500 (upward diagonal). The common *x*-axis label shows the percentage of propylene glycol. Results are shown as average ± SD (*n* = 6).

Formulation produced with varied polymer ratios (PUL: MTX = 300:100 mg) regardless of plasticizer amount, the D-time considerably raised as the proportion of MTX to PUL in film decreased and vice versa, as shown in Figure 10. This could be ascribed to the hydrophilic/oligosaccharide character of MTX, which altered polymer chain attrition that predominantly increased water infiltration to films, resulting in rapid disintegration. The findings are consistent with previous studies by El Meshad and El Hagrasy [36]. Furthermore, varied PG concentrations in four groups had a minimal effect on the D-time of the film. In addition, RZT-ODFs formed with PUL (single) had a higher D-time than MTX-containing films. The data shows that increases in film thickness significantly improved the D-time, as shown in Figure S3.

Table S8 shows the model summary for response D-time. A higher R^2^ value of 0.99 demonstrated that the model was significant, which defined 99% of variability around the mean. The pred-R^2^ of 0.9870 was in reasonable agreement with the Adj-R^2^ of 0.9924, i.e., the difference was less than 0.2. The significance of the model was proved by an F-value of 613.79 (Table S8). Moreover, the *p* < 0.0001 value specified that the model elements were significant, and there is only a 0.01% chance that a large F-value could occur due to noise.

The model for the dependent variable Y_7_ (D-time) created a multiple linear regression equation (Equation (13)).

(13) $$Y_{7}=+30+9.11{\;X}_{1}-4.81{\;X}_{2}-2.10X_{3}$$

**Figure 11:**
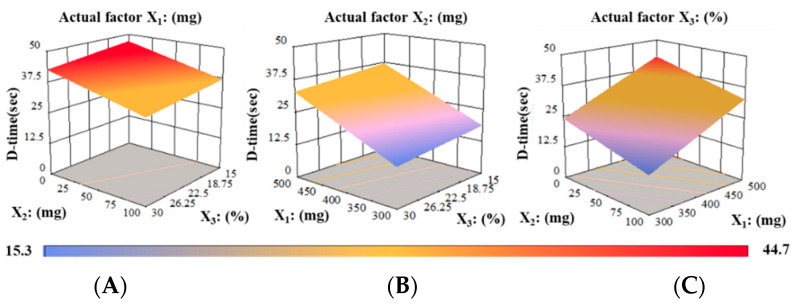
3D surface models describing the influence of (**A**) PUL (X_1_), (**B**) MTX (X_2_), and (**C**) PG (X_3_) on D-time (Y_7_) of RZT-ODFs.

A positive symbol in Equation (13) indicated that factor X_1_ positively impacted the independent parameter (Y_7_). A negative coefficient suggested that the factors (X_1_ and X_2_) were undesirably related to the independent response Y_2_. The findings showed that with the upsurge of PUL quantity (X_1_); the response Y_7_ (D-time) of RZT-ODFs extended, whereas MTX and PG (X_2_ and X_3_) portions reduced the D-time. The 3-D response surface model displays the influence of X_1_ (PUL), X_2_ (MTX), or X_3_ (PG) on Y_2_ (D-time) of RZT-ODFs. At consistent actual factor X_1_ (500 mg), with any amount of X_2_ (0–100 mg) and X_3_ (15–30%) showed a D-time ranging from 33.2 ± 2.3 to 44.7 ± 4.2 s, respectively (Figure 11A). Correspondingly, response Y_7_ increased as the amount of X_1_ increased; however, it decreased as the proportion of X_2_ and X_3_ in RZT-ODFs improved. A rapid D-time of 15.3 ± 3.1 was observed once the X_2_ level was held at a high level (100 mg), X_1_ at a low degree (300 mg), in a condition when X_3_ augmented to a high rate (30%) (Figure 11B). Moreover, Figure 11C clarified the substantial antagonistic and synergistic influence of X_1_ and X_2_ on the response D-time of RZT-ODFs. The findings coincide with the D-time linear regression developed by the statistical program.

### 3.5. In Vitro Dissolution Study

**Figure 12:**
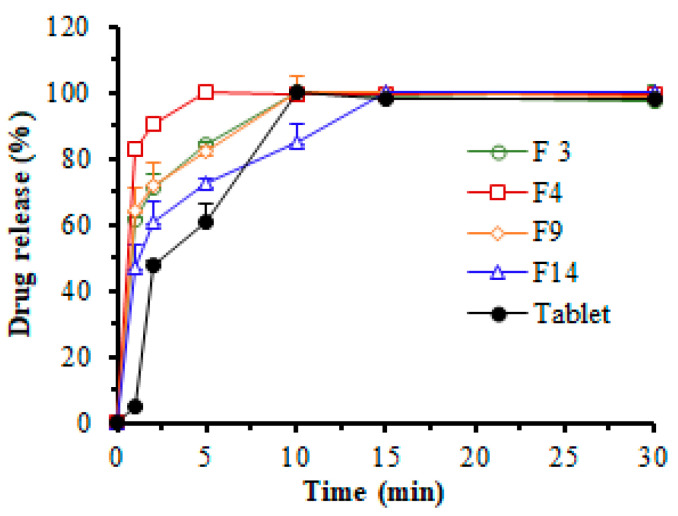
In vitro release pattern of RZT-ODFs and commercial tablets in simulated salivary fluid at pH 6.8. The various colored lines represent different formulations: green (F3; PUL = 300), dark red (F4; PUL:MTX = 300:100), orange (F9; PUL:MTX = 400:100), blue (F14; PUL:MTX = 500:100), and black color (commercial tablets). All RZT-ODFs were treated with the same amount of plasticizer (25%). Results are presented as a mean ± SD of triplicates.

The in vitro dissolution study of RZT-loaded ODFs was examined in an environment that mimicked mouth saliva with a pH of 6.8 (Figure 12). When a similar percentage of plasticizer (25%) was employed, the dissolution (%) of RZT-ODFs (t = 15 min) constituted of a lower amount of PUL (F3, 300 mg) was quicker compared to films composed of a higher amount of PUL (F14, 500 mg). This is attributed to the polymer’s wicking effect, which creates a stronger interlayer that delays water entry to the film, extending disintegration and dissolution. In contrast, RZT-ODFs with lower polymeric amounts rapidly dissolve and produce a porous channel that facilitates disintegration and drug dissolution [32,38,39]. All of the tested RZT-ODFs successfully released RZT after fifteen minutes. The release pattern discrepancy was noticed at the initial time intervals (2 to 15 min), as illustrated in Figure 12. During the first 15 min, the dissolving performance of RZT-ODFs with 100 mg MTX (F4) was substantially (*p* < 0.05) better compared to the formulation without MTX (F3). This is possibly due to the influence of oligosaccharide MTX on the PUL polymeric matrix, which increases water permeability and induces rapid disintegration of RZT-ODFs, simultaneously boosting solubilization and diffusion of RZT [36]. Furthermore, a significant (*p* < 0.05) faster RZT released was observed from the optimized film (F4) compared to the marketed product, attributed to hydrophilic polymer blends that dissolve rapidly in simulated saliva, which supports the loading of RZT to film-forming polymers.

### 3.6. Compatibility Test

**Figure 13:**
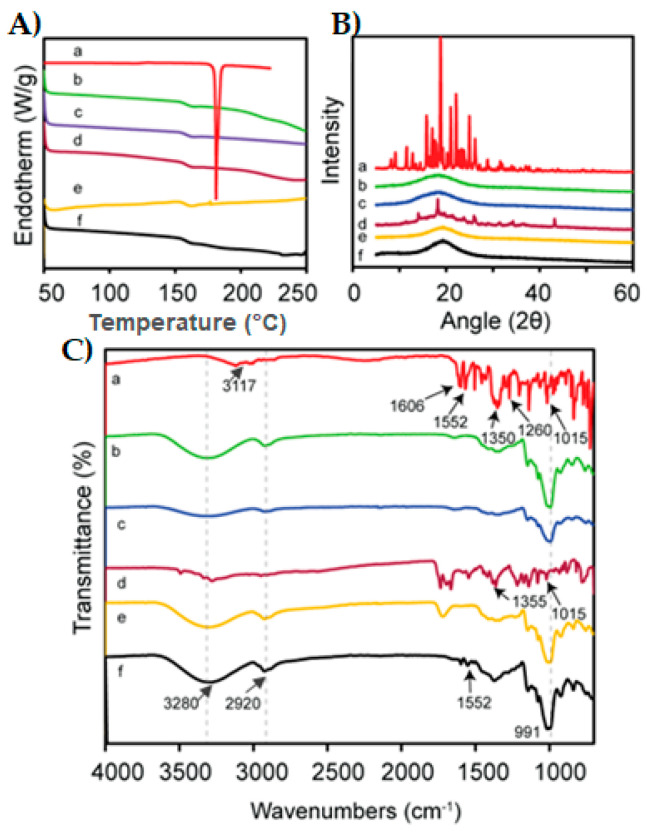
DSC thermo-grams (**A**), XRD patterns (**B**), and FTIR analysis (**C**) of pure RZT (a), MTX (b), PUL (c), physical mixture (d), blank-film (e), and RZT loaded film (f).

The thermal behavior of pure RZT, MTX, PUL, the corresponding physical mixture, blank film, and RZT-loaded ODFs was studied by DSC analysis, and their thermograms are shown in Figure 13A. The DSC thermogram of RZT exhibits a sharp endothermic peak at 177 °C, which corresponds to its melting point. Thus, the thermogram of RZT conferred its anhydrous and crystalline state [1]. No characteristic peak was detected in the thermogram of MTX, which might be due to its amorphous nature, and similar outcomes were proposed by Rania H et al. [40]. The peaks of crystallization or melting were not observed in the DSC thermogram of PUL, suggesting that PUL was amorphous. This might be because a steric interference of neighboring bulky side chains limits the rotation of glycosidic linkages in the PUL backbone, and these results agree with the reported ones [41]. While preparing the PM, partial disorientation of the organized crystalline structure of RZT occurs, which could justify by the low drug amount to film-forming components in the ternary PM system resulting in the lowest intensity endothermic peak in the DSC thermograph of PM, as previously reported [42]. In addition, no significant difference was observed in the blank film compared to the RZT-loaded film. The absence of a drug peak in the RZT-ODF thermogram suggested significant molecular miscibility and consistent drug distribution in film fabricating constituents [6].

XRD verified the crystallinity transformation of pure RZT in RZT-ODFs, as shown in Figure 13B. The presence of intense, sharp peaks at 15.8, 18.7, 20.9, 22.1, and 24.9 in the XRD diffract gram of pure RZT demonstrates that active RZT exists in pure crystalline form. The broad peak at 18.3 in the MTX and PUL XRD patterns confirmed the amorphous nature of polymers [31,43]. Some distinctive RZT peaks were observed in the physical mixture. Therefore, the hallo pattern of RZT-ODFs demonstrates the transition of all discrete peaks into a broad peak at 19.3, indicating the loss of crystallinity of RZT and its transformation into an amorphous state [44].

FTIR spectroscopy was performed to determine the functional groups and the typical vibrations of pure RZT and other excipients used in film formulation (Figure 13C). The pure RZT clearly showed characteristics peaks at 1563 cm^−1^, and 1606 cm^−1^ corresponding to C=O stretching. The most critical absorption peak appeared at 1372, attributed to C-N stretching in tertiary amines. C-O stretching in the carboxylic acid peak was noticed at 1295 cm^−1^ [45]. C-O and Hydrogen bond stretching was observed in the MTX IR spectrum at 1013 cm^−1^ and 991 cm^−1,^ respectively [46]. A strong absorption peak was observed in the PUL spectrum at 3297 cm^−1^, which indicates repeating units of –OH in PUL. Another strong peak at 2923 cm^−1^ and 846 cm^−1^ was attributed to the C-H bond of the alkane compound and α –D-glucopyranose configuration, respectively [47]. The existence of major RZT peaks and lack of shifting or generation of new peaks in the optimized film formulation (F4) confirmed the compatibility of RZT drugs with their excipients used for the formulation of RZT-ODFs [48].

### 3.7. Model Validation Using Desirability Function

The statistical validity of the polynomials was tested using the ANOVA features of the Design Expert software, as shown in Figure S9. Following that, feasibility and array analyses were used to determine the composition of optimal compositions. The contour plots were created using the Design-Expert software file formats. The formulation was optimized after completing polynomial equations for the dependent variables Y_1_, Y_2_, Y_3_, Y_4_, Y_5_, Y_6_, and Y_7_ with the factors X_1_, X_2_, and X_3_. The optimization analysis was used to determine the degree of independent variables (X_1_, X_2_, and X_3_) that would result in the highest value of desirability. The R^2^ values for all responses were determined to be 0.82 to 0.99, respectively. A low level of X_1_ (PUL = 300 mg), a high level of X_2_ (MTX = 100 mg), and a medium level of X_3_ (PG = 25%) were chosen for maximum desirability. The software generated formulation F4 (PUL:MTX = 300:100 mg::PG = 25%) as an optimized formulation which demonstrated rapid drug release after 5 min (100%), shortest D-time (16.0 s), and acceptable physicochemical and mechanical properties when compared to other formulations. Finally, repeated validating experiments were carried out utilizing the adjusted parameters to validate the data. The results were nearly similar to the data generated from optimization analysis employing desirability functions, suggesting that the BBD, combined with desirability functions, can be efficiently utilized for formulation optimization [49].

### 3.8. Animal Pharmacokinetic Performance

The plasma concentration of RZT in rats was evaluated using a highly reproducible, sensitive, and efficient HPLC approach that yielded two un-conjugated peaks of RZT and ZMT, employed as an internal reference (IS). The bio-analytical HPLC method was used for RZT analysis and produced a typical chromatogram for the analyte and IS (Figure S10).

**Figure 14:**
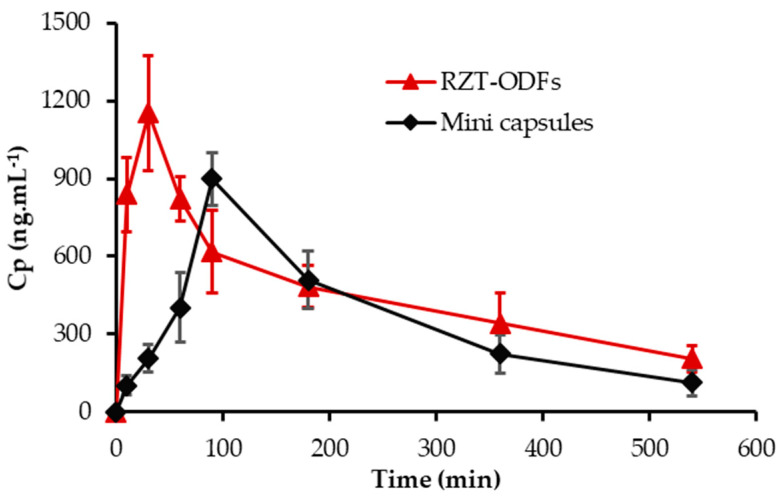
Pharmacokinetic profile of optimized RZT-ODFs and marketed product. Data expressed as average ± SD (*n* = 6).

The pharmacokinetic profile of the optimized formulation and the marketed product is shown in Figure 14. The optimized formulation (F4) was chosen for the comparative investigation versus the oral mini capsule as a reference to determine the pharmacokinetic characteristics of the tested formulation, as shown in Table 2. The average peak plasma concentrations for ODFs and mini-capsules were 1151.9 ± 223.1 ng·mL^−1^ and 898.2 ± 103.1 ng·mL^−1^, respectively. The C_max_ of the RZT-ODFs treated group was significantly (*p* ˂ 0.05) higher than that of the mini capsules treated group. The C_max_ of the RZT-ODFs treated group was significantly (*p* ˂ 0.05) higher than that of the mini capsules treated group. The findings showed that the time required to achieve peak plasma concentration (T_max_) of the experimental and commercial drugs was 0.5 and 1.5 hr, respectively. Furthermore, the mean area under the curve (AUC_0−t_) for RZT-ODFs and mini capsules were 4140.9 ± 630.0 and 3201.3 ± 467.9 ng·h·mL^−1^, respectively. The higher C_max_, AUC_0−t_, and lower T_max_ values of the optimized formulation shows a clear difference in absorption rates between experimental and commercial dosage forms because the ODFs bypass CYP-3A4 metabolism and GIT degradation of RZT, which is common with the conventional oral solid dosage form.

In addition, the drug is quickly released when it comes into contact with saliva in the oral mucosa owing to the enormous surface area of the RZT-ODFs. This leads to high absorption and penetration of the drug into the bloodstream [50]. Furthermore, no significant (*p* ˂ 0.05) variation in MRT between tested and reference formulations revealed that the RZT retention duration in vivo was approximately identical. The obtained results agree with previous studies [28,51]. However, the pharmacokinetic parameters of RZT-ODFs were only preliminary investigated in rats. Further in-depth and extensive investigations in other animals such as rabbits (lagomorph species), Beagle and other breed dogs are required to clarify pivotal pharmacokinetics parameters that could be relevant in a clinical context.

## 4. Conclusions

The optimized RZT-ODFs were fabricated using PUL:MTX::300:100 and PG as plasticizer (25%) by solvent casting method, revealing reasonable drug release kinetics (100%), in vitro D-time (16 s), and acceptable physiochemical and mechanical features. The formulated RZT-ODFs using the BBD were shown to be successful in generating statistically optimal composition with desirability function. The considerable variation in in vitro dissolution performance was evident compared to the marketed drug. Furthermore, RZT loaded in ODFs showed a significant improvement in in vivo performance compared to the marketed dosage form. Therefore, RZT-ODFs could help to treat migraines. Due to the prevalence of GIT dysfunction as a migraine symptom, RZT absorption is a severe issue. RZT-ODFs circumvent this by instantly entering the bloodstream, resulting in lower liver metabolism. The current work will likely give an excellent framework for anti-migraine therapeutic research and development.

## Figures and Tables

**Table 1:** Box–Behnken experimental design of three independent variables (X_1_, X_2_, and X_3_) and their respective influences on the corresponding responses (Y_1_, Y_2_, Y_3_, Y_4_, Y_5_, Y_6_ and Y_7_). All values are presented as mean ± SD (*n* = 3).

Code	PUL (mg, X_1_)	MTX (mg, X_2_)	PG (%, X_3_)	Thickness (Y_1_, µm)	FE (Y_2_, Folds)	TS (Y_3_, MPa)	Elongation (Y_4_, %)	Moisture Content (Y_5_, %)	Moisture Uptake (Y_6_, MPa)	D-Time (Y_7_, s)
F1	300	100	15	44.1 ± 5.8	64.7 ± 7.8	11.1 ± 3.5	14.0 ± 9.1	6.0 ± 1.7	5.4 ± 1.6	19.0 ± 1.8
F2	300	100	20	43.0 ± 5.3	80.7 ± 6.7	6.3 ± 4.0	25.4 ± 3.9	6.7 ± 1.9	5.7 ± 1.8	17.8 ± 1.6
F3	300	-	25	26.0 ± 4.5	121.3 ± 3.2	7.9 ± 0.9	28.4 ± 0.7	3.9 ± 0.9	2.1 ± 0.5	24.7 ± 4.1
F4	300	100	25	39.8 ± 5.0	110 ± 8.0	2.5 ± 0.1	58.9 ± 0.3	7.5 ± 2.1	5.8 ± 1.8	16.0 ± 2.4
F5	300	100	30	44.2 ± 4.6	156.3 ± 10.7	1.1 ± 0.9	62.2 ± 2.8	8.2 ± 2.4	6.1 ± 1.9	15.3 ± 3.1
F6	400	100	15	63.9 ± 7.4	88.7 ± 12.9	15.7 ± 0.4	13.9 ± 0.2	5.8 ± 1.8	4.4 ± 1.8	29.7 ± 2.9
F7	400	100	20	62.9 ± 6.8	112.7 ± 9.5	7.2 ± 4.0	24 ± 0.6	6.1 ± 2.5	4.5 ± 1.9	27.0 ± 1.7
F8	400	-	25	40.2 ± 5.3	142.3 ± 12.4	8.6 ± 8.1	27.7 ± 1.5	4.5 ± 1.3	2.6 ± 0.8	35.7 ± 3.0
F9	400	100	25	64.2 ± 4.5	128.3 ± 8.5	5.0 ± 4.0	53.2 ± 12.4	6.5 ± 2.8	4.7 ± 2.0	25.0 ± 2.0
F10	400	100	30	63.1 ± 4.6	193.0 ± 6.1	4.4 ± 1.0	54.8 ± 12.4	7.1 ± 3.0	4.8 ± 2.1	24.3 ± 2.0
F11	500	100	15	84.4 ± 5.0	106.3 ± 8.0	21.3 ± 1.8	13.4 ± 7.1	5.2 ± 2.9	3.5 ± 1.8	36.5 ± 3.3
F12	500	100	20	87.0 ± 6.6	146.3 ± 7.4	9.7 ± 5.9	21.1 ± 6.0	5.5 ± 3.1	3.7 ± 1.6	35.2 ± 3.9
F13	500	-	25	66.4 ± 5.5	173.0 ± 6.6	9.8 ± 9.4	25.2 ± 11.3	4.9 ± 2.2	2.8 ± 1.4	44.7 ± 4.2
F14	500	100	25	83.8 ± 4.1	160.7 ± 7.6	6.3 ± 2.7	42.1 ± 0.3	5.9 ± 3.3	4.0 ± 2.0	34.3 ± 2.5
F15	500	100	30	85.7 ± 5.0	243.3 ± 7.5	5.2 ± 3.9	51.1 ± 8.8	6.4 ± 3.6	4.5 ± 2.3	33.2 ± 2.3

**Table 2:** Pharmacokinetic parameters of optimized formulation and marketed product.

No.	PK-Parameters	RZT-ODFs	Mini Capsules
1	C_max_ (ng·mL^−1^)	1151.9 ± 223.1 *	898.2 ± 103.1
2	T_max_ (h)	0.5	1.5
3	AUC_(0−t)_ (ng·h·mL^−1^)	4140.9 ± 630.0 *	3201.3 ± 467.9
4	MRT (h)	3.3 ± 0.3	3.3 ± 0.3

* *p* ˂ 0.05 versus RZT mini capsules^®^ as a control.

## Data Availability

Not applicable.

## Supplementary Materials

The following supporting information can be downloaded at: https://www.mdpi.com/article/10.3390/pharmaceutics14122687/s1. Figure S1: Preparation process of RZT-ODFs using solvent casting method; Figure S2: Schematic diagram of blood sampling treatment for pharmacokinetics studies; Figure S3: Effect of polymeric material on the thickness and D-time of RZT-ODFs; Figure S4: Effect of plasticizer percentage on folding endurance; Figure S5: Effect of polymeric materials ratio on folding endurance; Figure S6: Surface pH values of RZT-ODFs; Figure S7: Effect of plasticizer level on the water content of RZT-ODFs; Figure S8: Effect of MDX concentration on moisture uptake of RZT-ODFs; Figure S9: Desirability ramp for numerical optimization of seven selected goals (A), over plot (B), and model desirability (C); Figure S10: Chromatograms of (a) blank plasma, (b) RZT, (c) zolmitriptan (IS), (d) RZT and IS; Table S1: Feasibility, pH and drug content (%) determination of RZT-ODFs; Table S2: Model summary and statistics (A), ANOVA for thickness (Y_1_) surface linear model (B); Table S3: Model summary and statistics (A), ANOVA for folding endurance (Y_2_), linear surface model (B); Table S4: Model summary, statistics and ANOVA for tensile strength (Y_3_) surface linear model; Table S5: Model summary and statistics and ANOVA for % E (Y_4_) surface linear model; Table S6: Model summary and statistics and ANOVA for water content (Y_5_) surface linear model; Table S7: Model summary and statistics and ANOVA for water absorption (Y_6_) surface linear model; Table S8: Model summary, statistics and ANOVA for D-time (Y_7_) surface linear model.
