# Supplementary material for: Rizatriptan-Loaded Oral Fast Dissolving Films: Design and Characterizations
Source: Pharmaceutics. 2022 Dec 1;14(12):2687. doi: 10.3390/pharmaceutics14122687 (PMC9780891; doi:10.3390/pharmaceutics14122687)
Supplement: Supplementary file 1 [file pharmaceutics-14-02687-s001.zip › pharmaceutics-1993919-supplementary.pdf]

Rizatriptan-loaded oral fast dissolving films: Design and characterization

Suplementry Materials

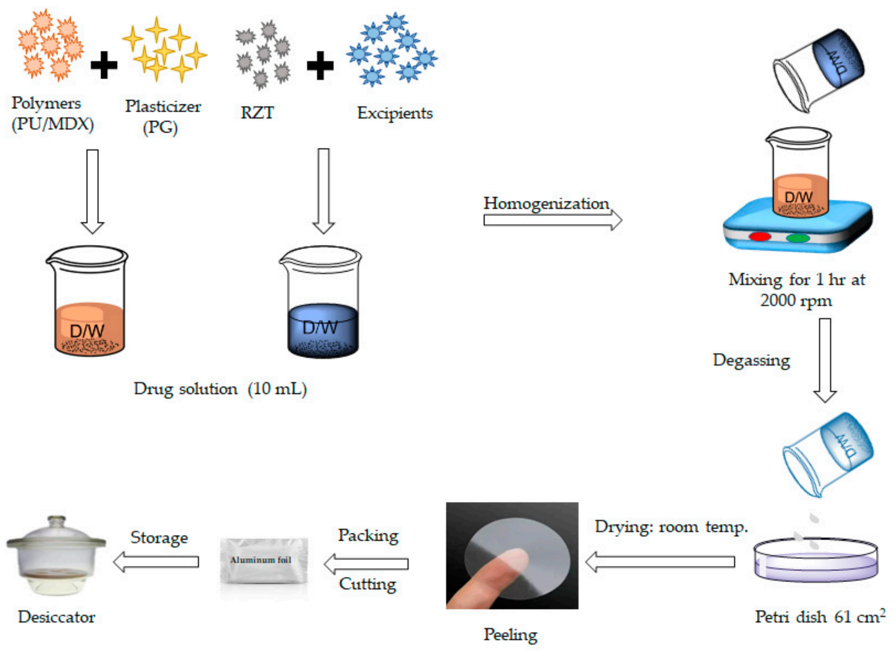

Figure S1. Preparation process of RZT-ODFs using solvent casting method.

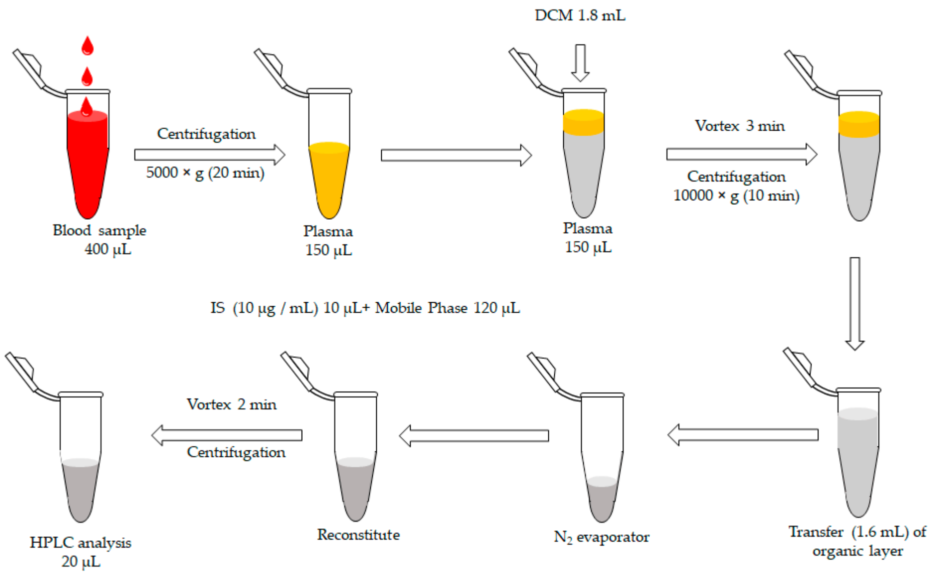

Figure S2. Schematic diagram of blood sampling treatment for pharmacokinetics studies

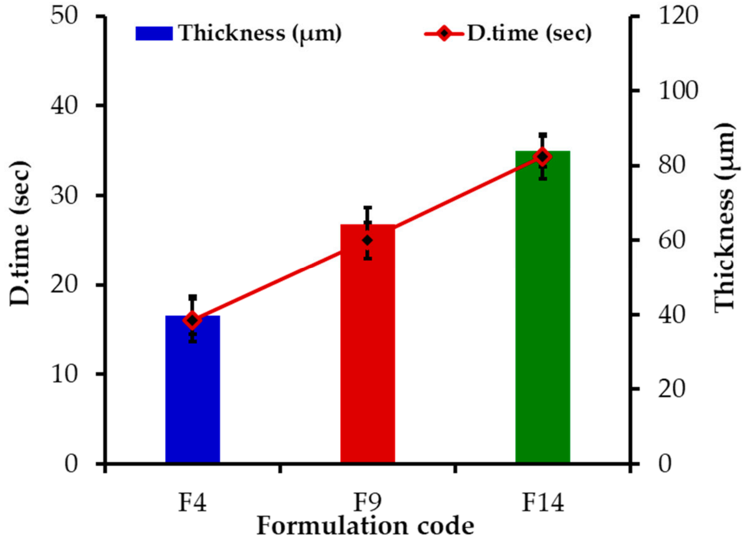

Figure S3. Effect of polymeric material on the thickness and D-time of RZT-ODFs

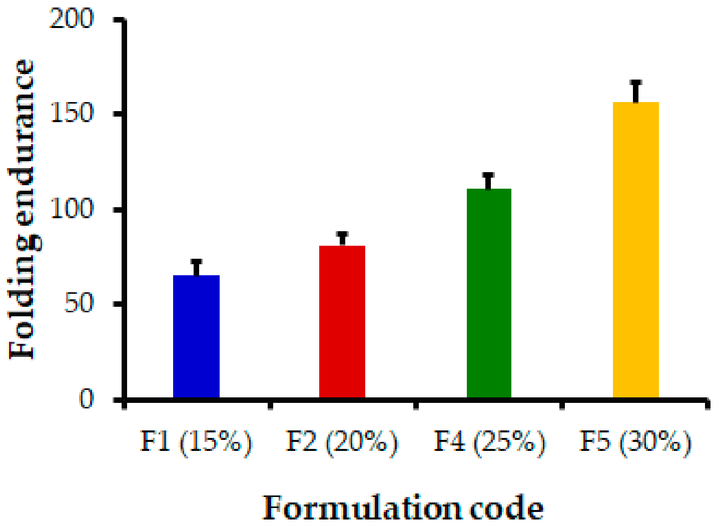

Figure S4. Effect of plasticizer percentage on folding endurance

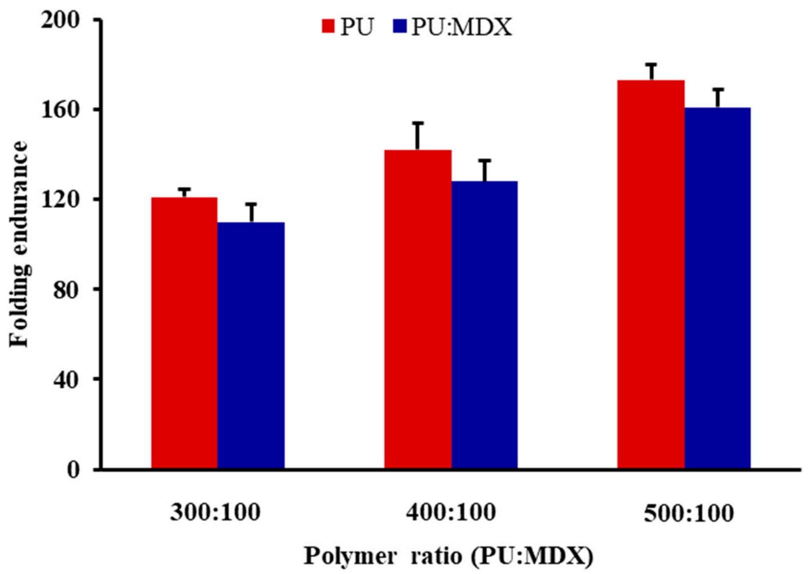

Figure S5. Effect of polymeric materials ratio on folding endurance

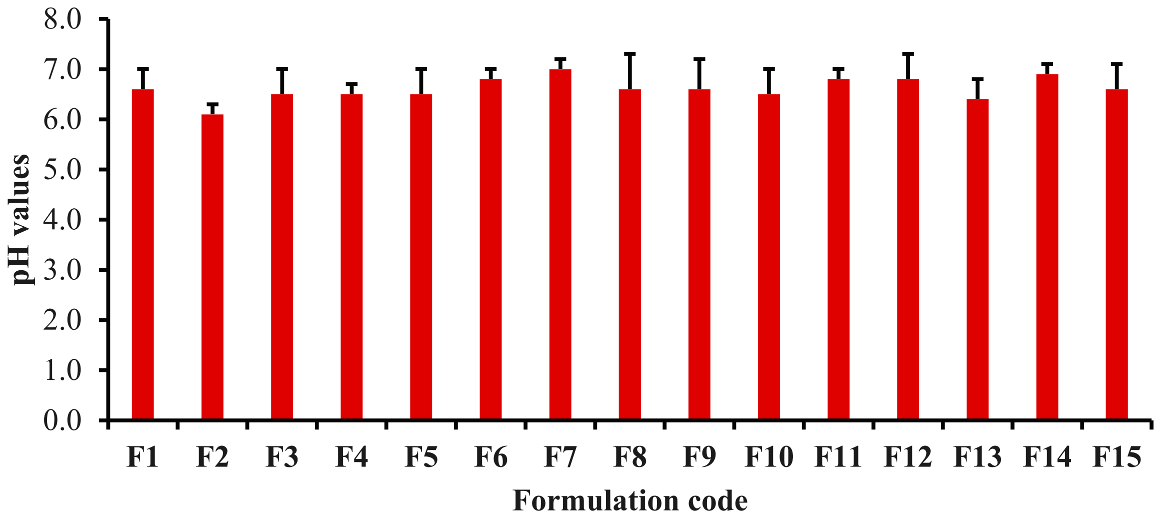

Figure S6. Surface pH values of RZT-ODFs

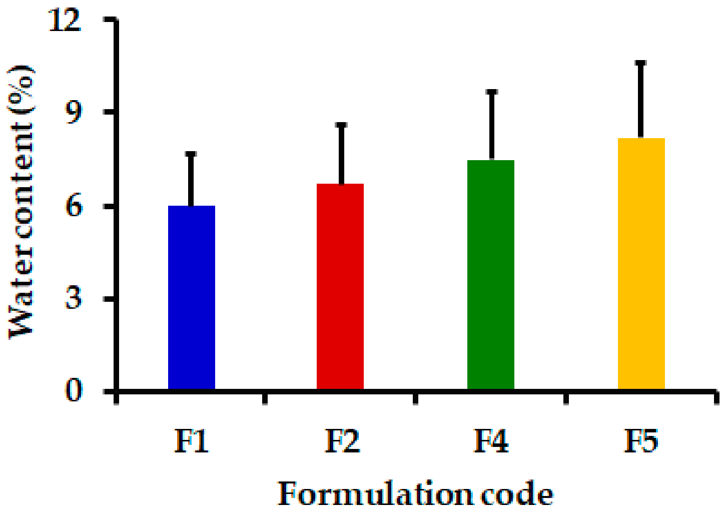

Figure S7. Effect of plasticizer level on the water content of RZT-ODFs

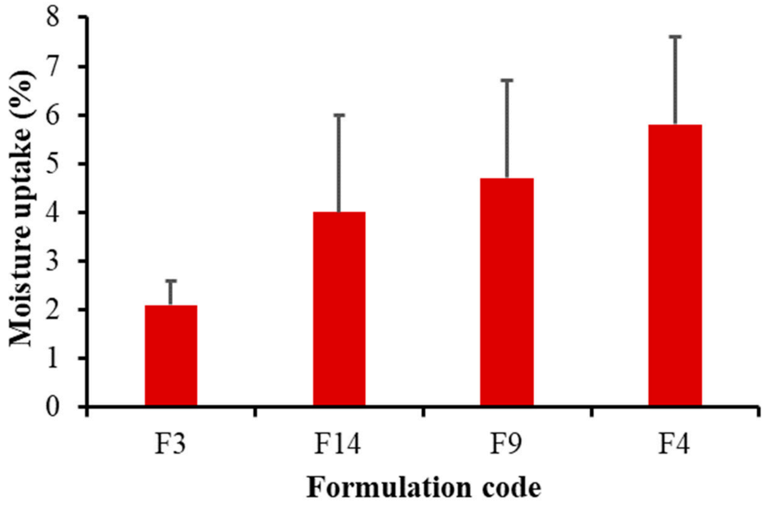

Figure S8. Effect of MDX concentration on moisture uptake of RZT-ODFs

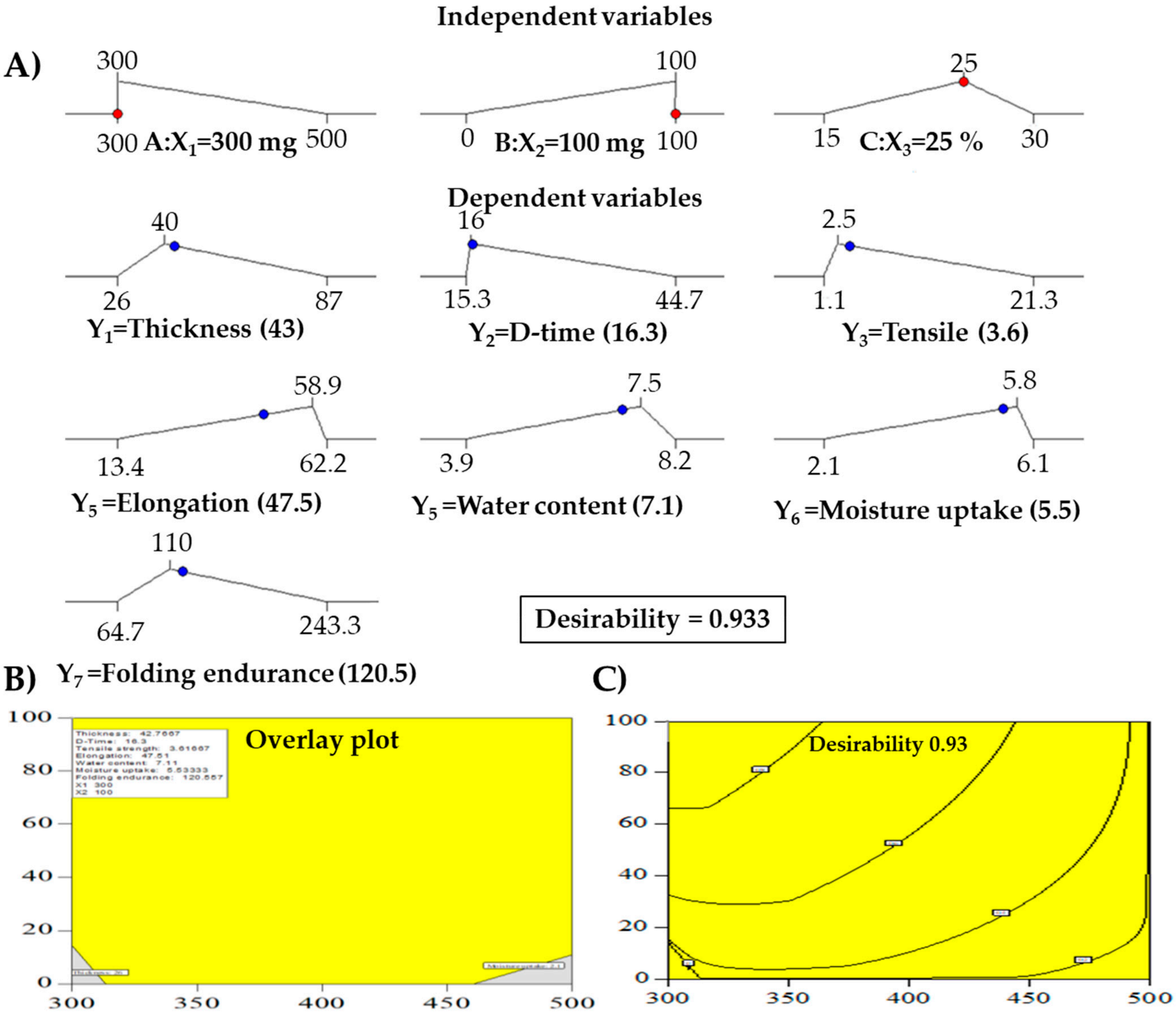

Figure S9. Desirability ramp for numerical optimization of seven selected goals (**A**), over plot (**B**), and model desirability (**C**).

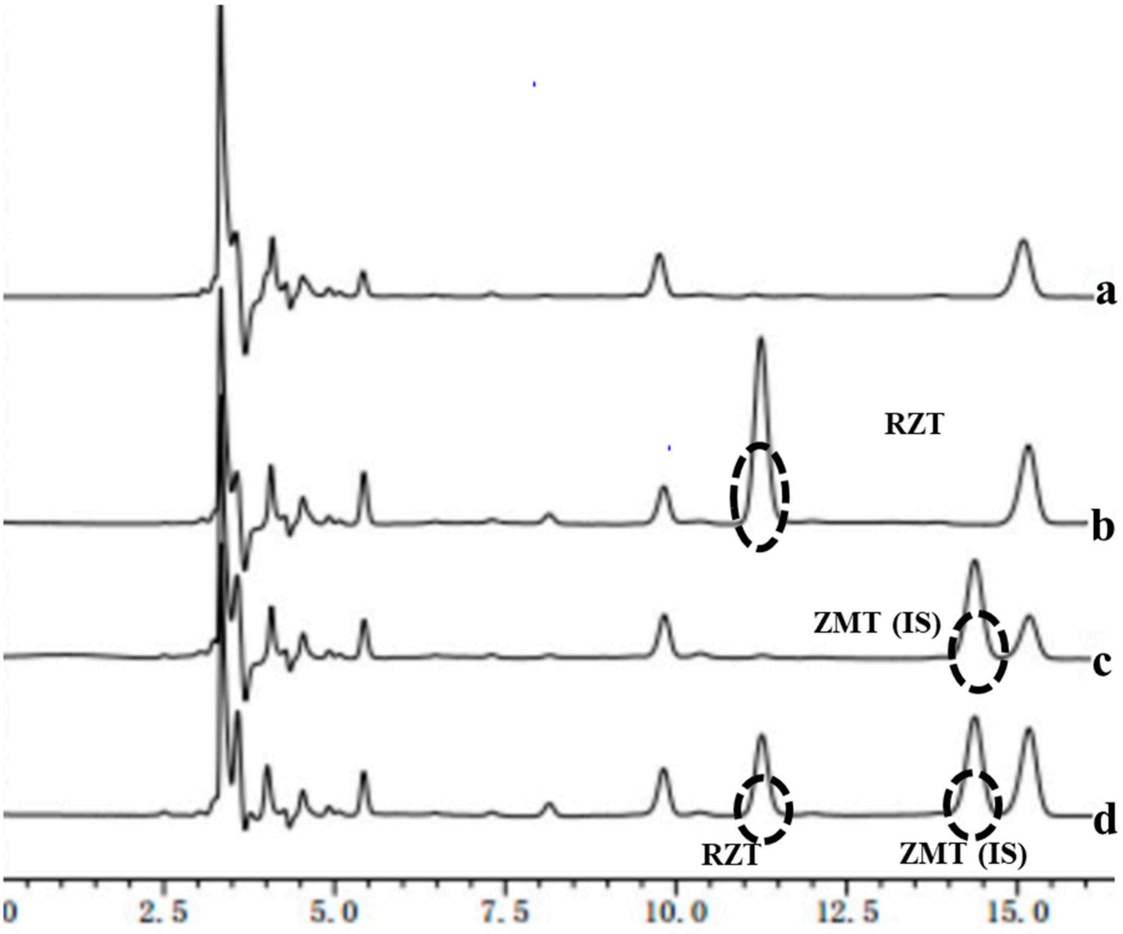

Figure S10. Chromatograms of (a) blank plasma, (b) RZT, (c) zolmitriptan (IS), (d) RZT and IS.

Table S1. Feasibility, pH and drug content (%) determination of RZT-ODFs.

Film Code	Adhesiveness	Film Clarity	Surface appearance	Drug content (%)	Weight (mg)	Av. pH ± SD
F1	Non-adhesive	Homogenous	Transparent	97.0±2.0	43.1±4.6	6.6±0.4
F2	Non-adhesive	Homogenous	Transparent	98.9±2.5	43.3±3.2	6.1±0.2
F3	Non-adhesive	Homogenous	Transparent	99.7±4.3	28.1±4.7	6.5±0.5
F4	Non-adhesive	Homogenous	Transparent	98.5±3.6	43.9±3.4	6.5±0.2
F5	Non-adhesive	Homogenous	Transparent	97.0±1.2	44.6±3.2	6.5±0.5
F6	Non-adhesive	Homogenous	Transparent	100.2±5.4	60.1±4.4	6.8±0.2
F7	Non-adhesive	Homogenous	Transparent	98.2±3.3	61.8±2.7	7.0±0.2
F8	Non-adhesive	Homogenous	Transparent	102.4±3.2	40.5±3.9	6.6±0.7
F9	Non-adhesive	Homogenous	Transparent	100.5±3.5	62.1±2.7	6.6±0.6
F10	Non-adhesive	Homogenous	Transparent	98.9±6.4	62.8±2.1	6.5±0.5
F11	Non-adhesive	Homogenous	Transparent	97.1±3.9	75.2±2.6	6.8±0.2
F12	Non-adhesive	Homogenous	Transparent	100.9±2.3	77.0±4.2	6.8±0.5
F13	Non-adhesive	Homogenous	Transparent	101.7±2.0	60.6±4.6	6.4±0.4
F14	Non-adhesive	Homogenous	Transparent	100.5±1.9	77.4±2.8	6.9±0.2
F15	Non-adhesive	Homogenous	Transparent	98.6±2.0	78.0±4.3	6.6±0.5

Table S2. Model summary and statistics (A), ANOVA for thickness (Y_1) surface linear model (B).

Model Summary and statistics of thickness (Y_1) Response							
Dependent variable	Model	Std. Dev.	R^2	Adj-R^2	Pred-R^2	Press	Statistical significance
Thickness (µm, Y_1)	Linear	2.05	0.99	0.99	0.9813	100.56	Suggested
	2FI	2.21	0.99	0.99	0.9291	381.93	Aliased
ANOVA for thickness (Y_1) response surface linear model							
Dependent variable	Source	Sum of squares	d.f.	Mean square	F-value	p-Value prob > F	Statistical significance
Thickness (µm, Y_1)	Model	5344.49	3	1781.50	425.61	< 0.0001	Significant
	X_1 (mg)	4418.40	1	4418.40	1055.57	< 0.0001	
	X_2 (mg)	885.32	1	885.32	211.51	< 0.0001	
	X_3 (%)	0.18	1	0.18	0.043	0.8389	

Table S3. Model summary and statistics (A), ANOVA for folding endurance (Y_2), linear surface model (B).

Model Summary and statistics of folding endurance (Y_2) Response

Dependent variable	Model	Std. Dev.	R^2	Adj-R^2	Pred-R^2	Press	Statistical significance
FE (Folds, Y_2)	Linear	14.11	0.9272	0.9073	0.8655	4039.84	Suggested
	2FI	14.08	0.9406	0.9076	0.8302	5102.60	Aliased

ANOVA for folding endurance (Y_2) response surface linear model

Dependent variable	Source	Sum of squares	d.f.	Mean square	F-value	p-Value prob > F	Statistical significance
FE (Folds, Y_2)	Model	27855.71	3	9285.24	46.67	< 0.0001	significant
	A-X1 (mg)	8797.16	1	8797.16	44.21	< 0.0001	
	B-X2 (mg)	50.62	1	50.62	0.25	0.6240	
	C-PG (%)	18656.07	1	18656.07	93.76	< 0.0001	

Table S4. Model summary, statistics and ANOVA for tensile strength (Y_3) surface linear model.

**Model Summary and statistics of TS (Y_3) Response**							
Dependent variable	Model	Std. Dev.	R^2	Adj-R^2	Pred-R^2	Press	Statistical Significance
Tensile strength (MPa, Y_3)	Linear	2.40	0.8268	0.7796	0.6655	122.46	Suggested
	2FI	2.37	0.8619	0.7851	0.5992	146.73	Aliased
ANOVA for TS (Y_3) response surface linear model							
Dependent variable	Source	Sum of squares	d.f.	Mean square	F-value	p-Value prob > F	Statistical Significance
Tensile strength (MPa, Y_3)	Model	302.67	3	100.89	17.50	0.0002	Significant
	X_1 (mg)	54.76	1	54.76	9.50	0.0104	
	X_2 (mg)	18.22	1	18.22	3.16	0.1030	
	X_3 (%)	246.44	1	246.44	42.76	< 0.0001	

Table S5. Model summary and statistics and ANOVA for % E (Y_4) surface linear model.

**Model Summary and statistics of % E (Y_4) Response**							
Dependent variable	Model	Std. Dev.	R^2	Adj-R^2	Pred-R^2	Press	Statistical Significance
Elongation (%, Y_4)	Linear	5.82	0.9131	0.8894	0.8613	594.84	Suggested
	2FI	5.91	0.9267	0.8860	0.8604	598.80	Aliased
ANOVA for % E (Y_4) response surface linear model							
Dependent variable	Source	Sum of squares	d.f.	Mean square	F-value	p-Value prob > F	Statistical Significance
Elongation (%, Y_4)	Model	3917.07	3	1305.69	38.53	< 0.0001	Significant
	X_1 (mg)	*129.60*	*1*	*129.60*	*3.82*	*0.0764*	
	X_2 (mg)	*652.10*	*1*	*652.10*	*19.24*	*0.0011*	
	X_3 (%)	*3589.81*	*1*	*3589.81*	*105.93*	*< 0.0001*	

Table S6. Model summary and statistics and ANOVA for water content (Y_5) surface linear model.

Model Summary and statistics of water content (Y_5) Response							
Dependent variable	Model	Std. Dev.	R^2	Adj-R^2	Pred-R^2	Press	Statistical significance
Water content (%, Y_5)	Linear	0.49	0.8550	0.8155	0.6040	7.13	Suggested
	2FI	0.11	0.9937	0.9902	0.9797	0.37	Aliased

ANOVA for water content (Y_5) response surface linear model

Dependent variable	Source	Sum of squares	d.f.	Mean square	F-value	p-Value prob > F	Statistical significance
Water content (%, Y_5)	Model	15.41	3	5.14	21.63	< 0.0001	significant
	X_1 (mg)	1.94	1	1.94	8.15	0.0156	
	X_2 (mg)	11.54	1	11.54	48.62	< 0.0001	
	X_3 (%)	4.11	1	4.11	17.30	0.0016	

Table S7. Model summary and statistics and ANOVA for water absorption (Y_6) surface linear model.

Model Summary and statistics of water absorption (Y_6) Response							
Dependent variable	Model	Std. Dev.	R^2	Adj-R^2	Pred-R^2	Press	Statistical significance
Moisture absorption (%, Y_6)	Linear	0.51	0.8596	0.8213	0.6139	7.81	Suggested
	2FI	0.17	0.9872	0.9801	0.9533	0.94	Aliased

ANOVA for water absorption (Y_6) response surface linear model

Dependent variable	Source	Sum of squares	d.f.	Mean square	F-value	p-Value prob > F	Statistical significance
Moisture absorption (%, Y_6)	Model	17.39	3	5.80	22.45	< 0.0001	significant
	X_1 (mg)	4.36	1	4.36	16.87	0.0017	
	X_2 (mg)	13.00	1	13.00	50.35	< 0.0001	
	X_3 (%)	0.79	1	0.79	3.07	0.1073	

Table S8. Model summary, statistics and ANOVA for D-time (Y_7) surface linear model.

Model Summary and statistics of D-time (Y_7) Response							
Dependent variable	Model	Std. Dev.	R^2	Adj-R^2	Pred-R^2	Press	Statistical significance
D-time (Sec, Y_7)	Linear	0.76	0.9941	0.9924	0.9870	13.74	Suggested
	2FI	0.68	0.9960	0.9938	0.9838	17.12	Aliased

ANOVA for D-time (Y_7) response surface linear model							
Dependent variable	Source	Sum of squares	d.f.	Mean square	F-value	p-Value prob > F	Statistical significance
D-time (Sec, Y_7)	Model	1050.35	3	350.12	613.79	< 0.0001	Significant
	X_1 (mg)	829.92	1	829.92	1454.92	< 0.0001	
	X_2 (mg)	213.71	1	213.71	374.65	< 0.0001	
	X_3 (%)	29.26	1	29.26	51.30	< 0.0001	
